# Iron Deficiency Reduces Cadmium Translocation in Peanut by Increasing the Root Cell Wall Reservoir

**DOI:** 10.3390/plants15172641

**Published:** 2026-08-28

**Authors:** Rui Liu, Jiaqi Ma, Qiyue Zhang, Gangrong Shi

**Affiliations:** College of Life Sciences, Huaibei Normal University, Huaibei 235000, China; 12311070721@chnu.edu.cn (R.L.); 12311070722@chnu.edu.cn (J.M.); 12411070178@chnu.edu.cn (Q.Z.)

**Keywords:** cadmium, iron deficiency, cell wall remodeling, peanut, root retention

## Abstract

Iron (Fe) deficiency and cadmium (Cd) contamination often co-occur in agricultural systems, yet the way in which Fe deficiency modulates Cd translocation remains unclear. Here, we investigated root cell wall modifications mediating Cd accumulation in two peanut cultivars with contrasting Fe deficiency tolerance. Fe deficiency significantly increased root Cd concentrations in both cultivars but reduced Cd translocation to shoots, an effect more pronounced in the tolerant cultivar Silihong. Cell wall analysis revealed cultivar-specific compositional changes: pectin and cellulose increased under combined Cd exposure and Fe deficiency, while hemicellulose (HC1) decreased. Negative correlations between Fe and Cd accumulation in roots, cell walls, and their components indicate competition between these two metal ions for binding sites in root cell walls. Increased pectin content under combined stress enhances Cd sequestration, while reduced HC1 content facilitates Fe mobilization to shoots. Transcriptomic analysis identified hub genes associated with cell wall modification, including pectinesterases (*PME2/4/29/63*), beta-galactosidases (*BGAL3/5/8*), polygalacturonases (*PG*s), pectin acetylesterases (*PAE8*), xyloglucan endotransglucosylase/hydrolases (*XTH8/31*) and laccases (*LAC7/11/15*). Under combined stress, Silihong exhibited superior Cd immobilization, characterized by higher Cd accumulation in HC1 and cellulose fractions, stronger induction of *PME*, *PAE8* and *LAC* genes, and greater suppression of *XTH*s, *PG*s, and *BGAL*s. Our findings demonstrate that Fe deficiency restricts Cd translocation by remodeling root cell walls, increasing pectin and cellulose while modulating hemicellulose integrity, thereby creating an expanded apoplastic reservoir that traps Cd. This structural detoxification mechanism, operating downstream of uptake transporters, identifies key cell wall components and regulatory genes as potential targets for breeding peanut cultivars with improved food safety.

## 1. Introduction

Cadmium (Cd) is a non-essential and highly toxic heavy metal that has become a major environmental contaminant, primarily due to anthropogenic activities such as industrial emissions, mining, and the application of phosphate fertilizers and sewage sludge to agricultural land [1,2]. Its high mobility in the soil–plant system facilitates uptake by crop roots and subsequent accumulation in edible tissues. Dietary intake of Cd-contaminated foods is the primary route of human exposure, leading to serious health risks including renal dysfunction, skeletal damage (as tragically illustrated by the historic “Itai-itai” disease), and various cancers [1]. These concerns have made Cd a critical priority for food safety regulators worldwide.

Iron (Fe) deficiency is one of the most widespread micronutrient disorders affecting global crop production, particularly in calcareous and alkaline soils where Fe bioavailability is low [3]. As an essential element, Fe serves as a cofactor for numerous enzymes and is integral to key biological processes such as chlorophyll synthesis, photosynthesis, and respiration. Iron deficiency in plants typically results in interveinal chlorosis of young leaves, stunted growth, and significant losses in both yield and nutritional quality [4]. This agricultural challenge has a direct human health corollary: Fe deficiency anemia affects nearly 2 billion people [5], and consumption of Fe-poor staple crops exacerbates this global health burden.

Critically, Cd contamination and Fe deficiency often co-occur in agricultural systems, creating a complex physiological interaction in plants. Cd exposure can decrease Fe concentrations and induce severe chlorosis in various species, including rice (*Oryza sativa*) [6], maize (*Zea mays*) [7], wheat (*Triticum aestivum*) [7], and apple tree (*Malus xiaojinensis*) [8]. Conversely, Fe deficiency has been shown to enhance Cd uptake and accumulation across a wide range of crops, such as rice [9], maize [7], wheat [7], amaranth (*Amaranthus mangostanus*) [10], pea (*Pisum sativum*) [11], and black nightshade (*Solanum nigrum*) [12]. This bidirectional interaction underscores the need to understand the molecular and physiological crosstalk between Fe and Cd.

Owing to its similar ionic hydrated radius and chemical properties to Fe^2+^, Cd^2+^ is thought to enter plant cells and undergo translocation via Fe^2+^ transporters, competing with Fe for transport. Several Fe^2+^ transporters have been shown to mediate Cd uptake in roots, including AtIRT1 in *Arabidopsis* [13], as well as OsIRT1, OsIRT2, and OsNRAMP1 in rice [9,14]. Similarly, the Mn/Fe transporter OsNRAMP5 plays a central role in Cd uptake from soil in rice [15,16]. In addition, members of the heavy metal-transporting ATPase family, including OsHMA2 and OsHMA3, have been implicated in Cd translocation and vacuolar sequestration [17,18,19,20]. Collectively, these findings highlight the central role of essential metal transporters in Cd accumulation.

Besides membrane transporters, the cell wall may also play a critical role in determining Cd fate. As the outermost layer of plant cells, the cell wall serves as the primary interface for interaction with metal ions. It is predominantly composed of polysaccharides, including cellulose, hemicellulose, and pectin, which provide abundant metal-binding sites through negatively charged groups. Among these, hemicellulose and/or pectin have been proven to be the major polysaccharide responsible for Cd binding in root cell walls [21,22,23,24]. To counteract Cd toxicity, plants can modify their cell wall composition, thereby increasing Cd retention capacity [25,26]. In addition to immobilizing Cd, the cell wall also acts as a Fe pool that can be remobilized under Fe-limited conditions [27,28]. The alleviation of Fe deficiency by NaCl, NO, potassium (K), and NH_4_^+^ has been attributed to enhanced Fe reutilization facilitated by cell wall modifications [29,30,31,32]. Moreover, phosphorus deficiency-induced reduction in hemicellulose content promotes Fe reutilization and reduces Cd adsorption in the root cell wall, thereby mitigating both Fe deficiency and Cd toxicity [33,34]. These studies suggest that the cell wall is a dynamic structure that responds to nutrient stress and influences metal ion partitioning. Despite this progress, it remains unclear how Fe deficiency directly affects the structural and compositional properties of root cell walls, and what role these alterations play in modulating Cd uptake and translocation under Fe-deficient conditions.

Peanut (*Arachis hypogaea* L.) is an economically important legume crop worldwide, highly valued for its oil and protein content. It is not only sensitive to Fe deficiency [35] but also exhibits a high capacity for accumulating Cd in both its seeds and vegetative tissues [36,37]. Compounding this issue, Fe deficiency has been shown to significantly increase the uptake and accumulation of Cd in peanut plants [38]. Even though several gene families, such as *AhNRAMP* [39] and *AhOPT* [40], have been confirmed to participate in Fe/Cd interactions in peanut, the underlying mechanisms by which the cell wall governs Cd uptake and translocation under Fe-deficient conditions remain largely unknown.

To address this knowledge gap, the current study investigated the role of root cell wall modifications in mediating Cd accumulation and translocation in two peanut cultivars differing in Fe deficiency tolerance. We hypothesized that Fe deficiency triggers biochemical modifications to root cell wall components that enhance Cd sequestration in the apoplast, thereby restricting Cd translocation to shoots. The specific objectives were to: (1) quantify the effects of Fe deficiency on Cd uptake, accumulation, and organ-specific translocation; (2) compare cell wall component contents under varying Fe and Cd regimes; (3) link Fe deficiency-induced cell wall modifications to enhanced Cd sequestration and reduced Cd translocation; and (4) identify key genes involved in cell wall modifications. The findings are expected to reveal a structural detoxification mechanism and inform breeding strategies for peanuts with improved root-based phytostabilization capacity, contributing to safer food production.

## 2. Results

### 2.1. Influence of Fe Deficiency and Cd Exposure on Growth of Two Peanut Cultivars

**Figure 1:**
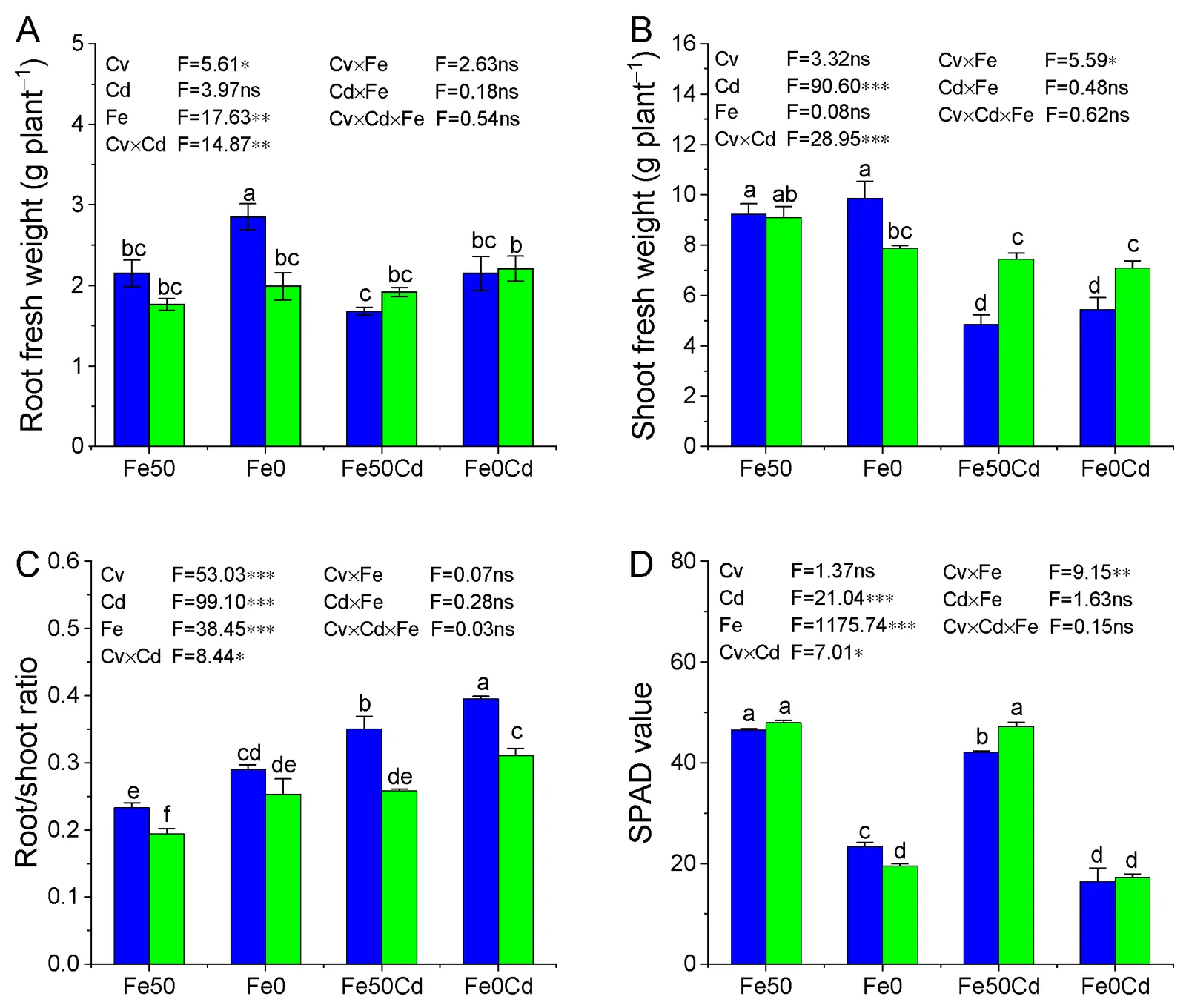
Effects of iron deficiency and cadmium stress on root fresh weight (**A**), shoot fresh weight (**B**), root/shoot ratio (**C**), and leaf chlorophyll content (SPAD value, (**D**)) in two peanut cultivars, Silihong (blue bars) and Fenghua 1 (green bars). Seedlings were grown under Fe-sufficient (50 μM of Fe-EDTA) or Fe-deficient (0 μM of Fe-EDTA) conditions with or without 2 μM of CdCl_2_ for 14 days. Values are means ± SE (*n* = 3). Bars sharing the same letter(s) are not significantly different at the 0.05 level according to Duncan’s multiple range test. Cv, cultivar; * *p* < 0.05, ** *p* < 0.01, and *** *p* < 0.001; ns, not significant.

Under iron deficiency, root fresh weight increased in Silihong but remained unchanged in Fenghua 1, while shoot fresh weight was not affected in either cultivar, resulting in an increased root/shoot ratio (Figure 1A–C). Exposure to 2 μM CdCl_2_ alone significantly reduced shoot fresh weight in both cultivars without affecting root fresh weight, also leading to elevated root/shoot ratios (Figure 1A–C). A similar pattern was observed under combined iron deficiency and cadmium stress, where shoot fresh weight decreased markedly in both cultivars while root fresh weight remained unaffected, again resulting in increased root/shoot ratios (Figure 1A–C).

Iron deficiency significantly reduced leaf chlorophyll content (SPAD value) in both cultivars, with a more pronounced decrease in Fenghua 1 than in Silihong (Figure 1D). Cadmium treatment alone decreased SPAD values in Silihong but had no significant effect in Fenghua 1. Under combined iron deficiency and cadmium exposure, SPAD values were dramatically reduced in both cultivars (Figure 1D).

### 2.2. Influence of Fe Deficiency on Cadmium Accumulation in Two Peanut Cultivars

**Figure 2:**
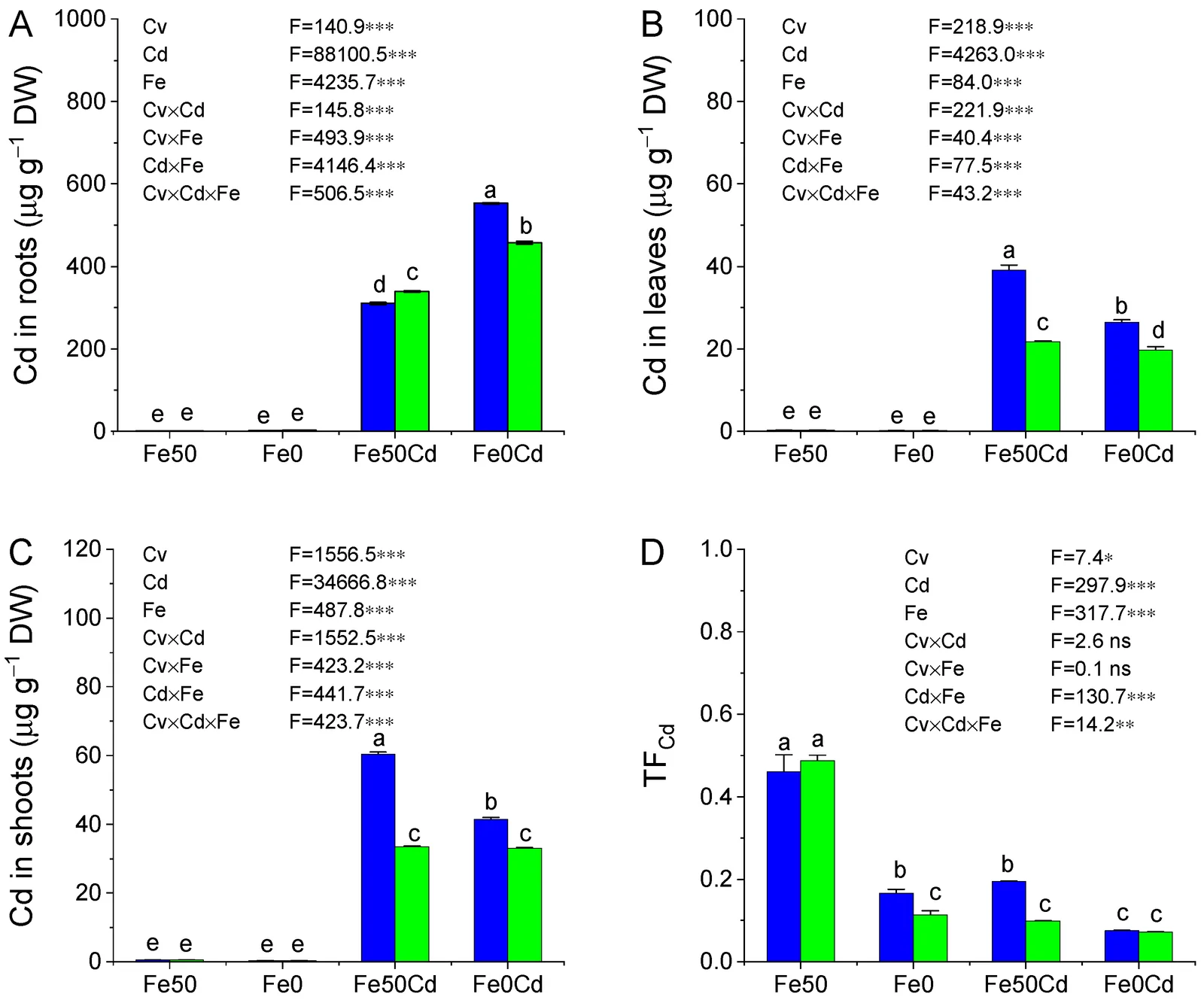
Cd concentrations in roots (**A**), leaves (**B**), shoots (**C**), and translocation factors (TF, (**D**)) of Fe-sufficient (Fe50) or Fe-deficient (Fe0) plants of Silihong (blue columns) and Fenghua 1 (green columns) exposed to 0 or 2 μM of CdCl_2_ for 14 days. Data (means ± SE, *n* = 3) sharing the same letter(s) above the error bars are not significantly different at the 0.05 level according to Duncan’s multiple range test. Cv, cultivar; * *p* < 0.05, ** *p* < 0.01, and *** *p* < 0.001; ns, not significant.

The two peanut cultivars exhibited distinct Cd accumulation patterns, which were strongly modulated by Fe deficiency (Figure 2). Under Fe-sufficient conditions, Silihong accumulated lower Cd concentrations in roots than Fenghua 1; however, under Fe deficiency, root Cd concentrations were higher in Silihong. In contrast, regardless of Fe supply, Cd concentrations in leaves and shoots remained consistently higher in Silihong than in Fenghua 1 (Figure 2A–C). A significant cultivar × Fe supply interaction was observed for Cd concentrations in roots, leaves, and shoots (Figure 2A–C), indicating that the effect of Fe deficiency on Cd accumulation was cultivar-dependent. Specifically, Fe deficiency increased Cd concentrations in roots but reduced them in leaves and shoots, with these effects being more pronounced in Silihong than in Fenghua 1. The translocation factor of Cd from roots to shoots decreased under Cd exposure and was further reduced by Fe deficiency, particularly in Silihong (Figure 2D).

### 2.3. Influence of Fe Deficiency and Cd Exposure on Fe Accumulation in Two Peanut Cultivars

**Figure 3:**
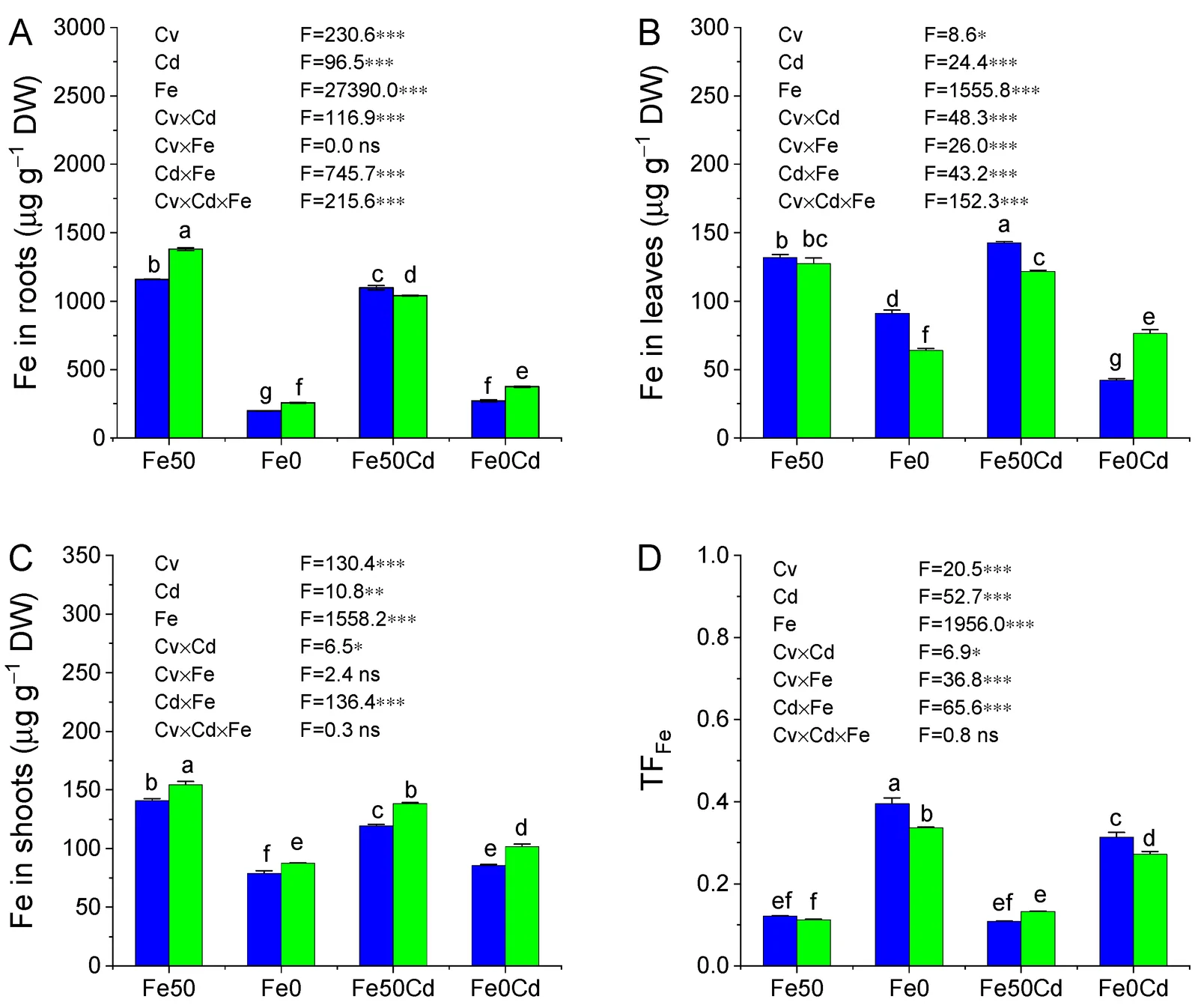
Fe concentrations in roots (**A**), leaves (**B**), shoots (**C**), and translocation factors (TF, (**D**)) of Fe-sufficient (Fe50) or Fe-deficient (Fe0) plants of Silihong (blue columns) and Fenghua 1 (green columns) exposed to 0 or 2 μM of CdCl_2_ for 14 days. Data (means ± SE, *n* = 3) sharing the same letter(s) above the error bars are not significantly different at the 0.05 level according to Duncan’s multiple range test. Cv, cultivar; * *p* < 0.05, ** *p* < 0.01, and *** *p* < 0.001; ns, not significant.

Iron accumulation in plant tissues and the corresponding root-to-shoot translocation factors were significantly affected by cultivar, Cd exposure, and Fe supply, as well as by the interactions between cultivar and Cd exposure and between Cd exposure and Fe supply (Figure 3). Regardless of Cd exposure, Fe deficiency markedly reduced Fe accumulation but enhanced its root-to-shoot translocation in both cultivars. However, the reduction in leaf Fe concentration was less in Silihong (30.8%) than in Fenghua 1 (49.8%) (Figure 3B).

Under Fe-sufficient conditions, Cd exposure significantly decreased Fe accumulation in both cultivars, while it increased the Fe translocation factor in Fenghua 1. Under Fe-deficient conditions, Cd exposure increased Fe concentrations in roots and shoots, as well as the Fe translocation factor, in both cultivars. Notably, leaf Fe concentration increased in response to Cd exposure in Fenghua 1 but decreased in Silihong (Figure 3B).

### 2.4. Correlation of Cd and Fe Accumulation in Plants

**Figure 4:**
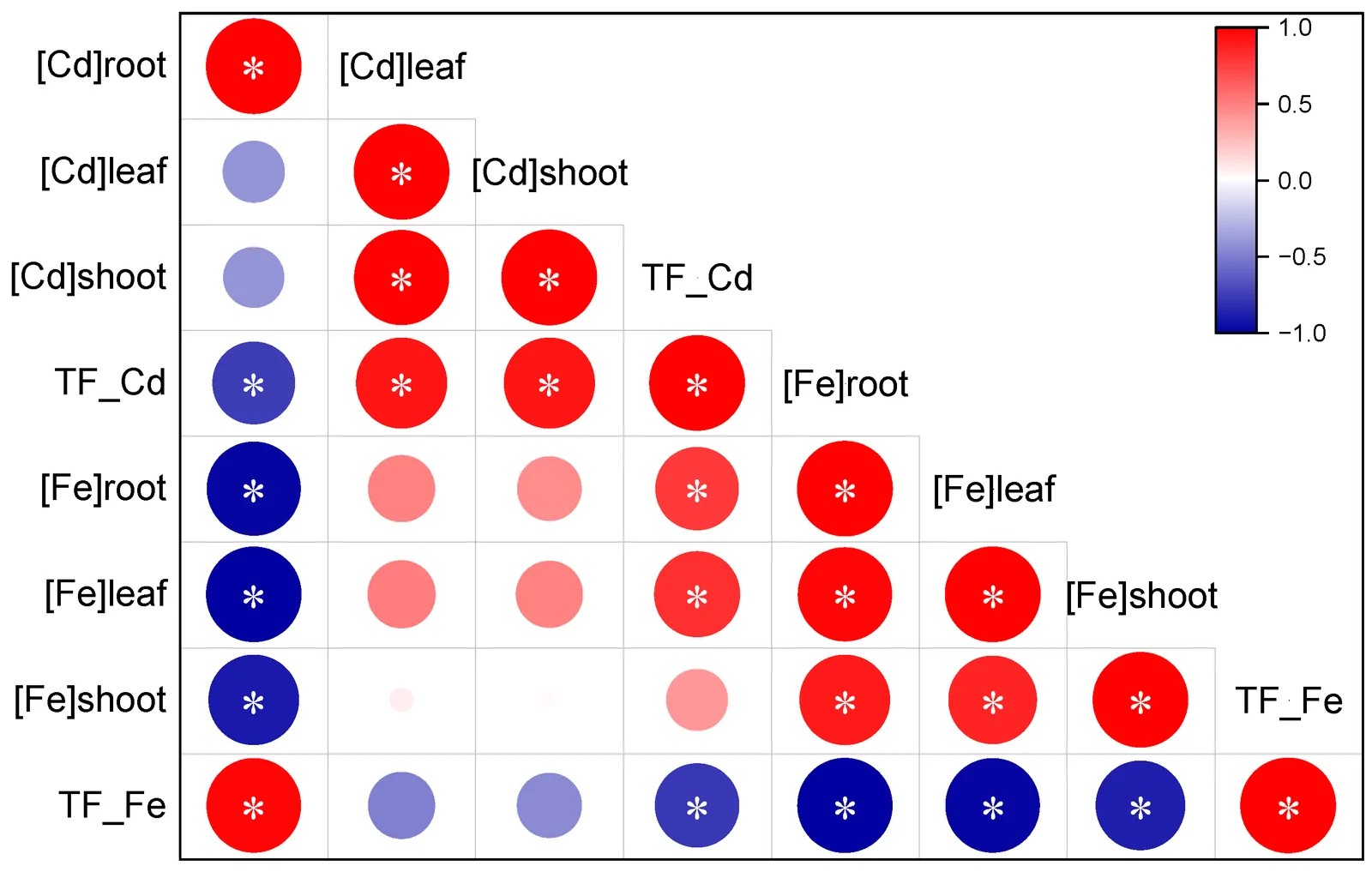
Correlation matrix of Cd and Fe accumulation in the peanut cultivars Silihong and Fenghua 1. Asterisks (*) indicate significant correlations at *p* < 0.05.

The correlation matrix revealed that Cd translocation factors were positively correlated with Cd concentrations in leaves and shoots, but negatively correlated with root Cd concentrations (Figure 4). Fe concentrations in roots, leaves, and shoots were positively correlated with one another, and all showed negative correlations with Fe translocation factors. Notably, Cd concentrations in roots negatively correlated with Fe concentrations in roots, shoots and leaves, but positively correlated with root-to-shoot Fe translocation factors. A negative correlation was also observed between the translocation factors Cd and Fe, suggesting an antagonistic relationship between iron and cadmium transport pathways.

### 2.5. Influence of Fe Deficiency and Cd Exposure on Root Cell Wall Component

**Figure 5:**
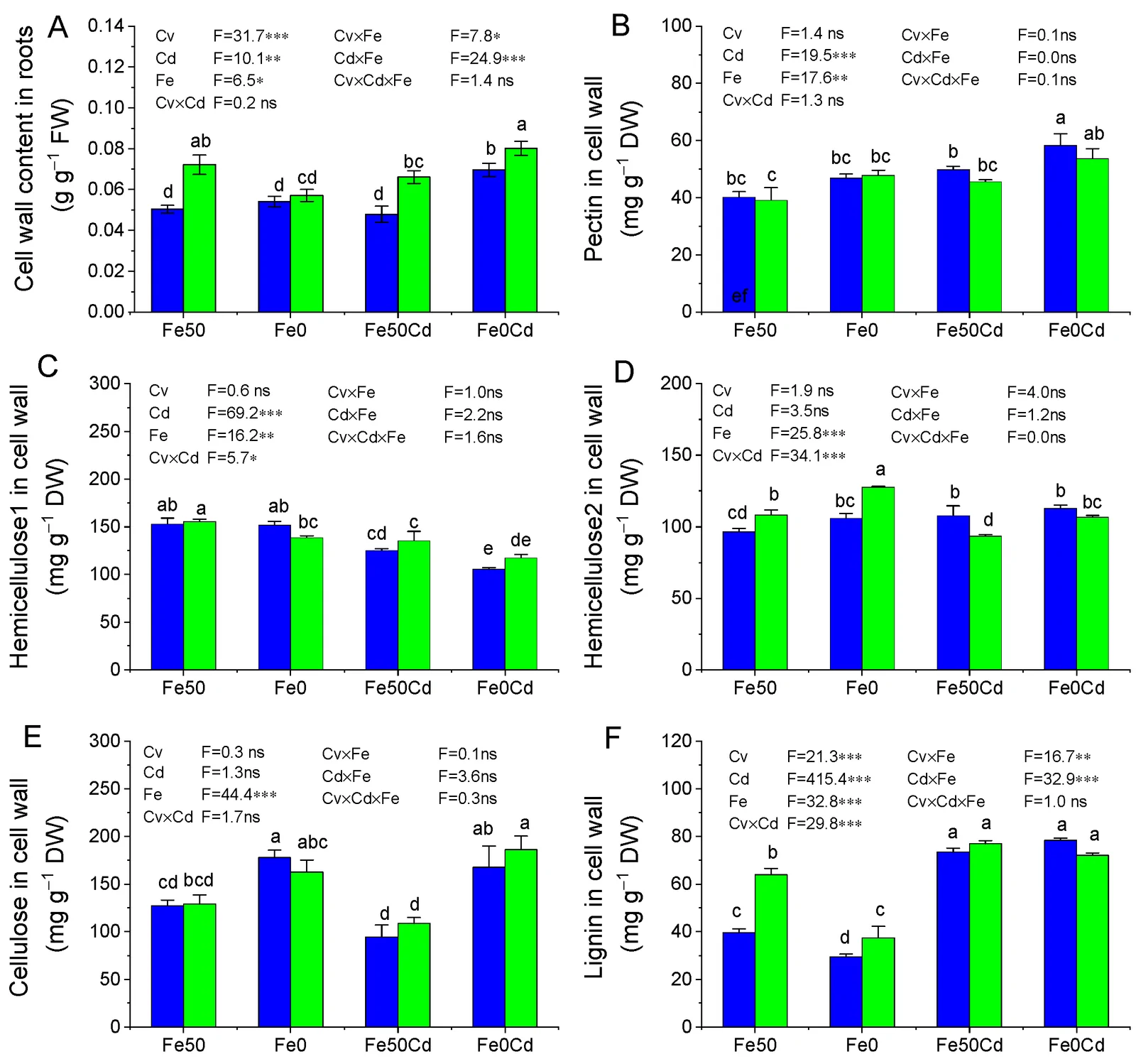
Cell wall content in roots (**A**), and the contents of pectin (**B**), hemicellulose 1 (**C**), hemicellulose 2 (**D**), cellulose (**E**), and lignin (**F**) in cell wall of Fe-sufficient (Fe50) or Fe-deficient (Fe0) plants of Silihong (blue columns) and Fenghua 1 (green columns) exposed to 0 or 2 μM of CdCl2 for 14 days. Data (means ± SE, *n* = 3) sharing the same letter(s) above the error bars are not significantly different at the 0.05 level according to Duncan’s multiple range test. Cv, cultivar; * *p* < 0.05, ** *p* < 0.01, and *** *p* < 0.001; ns, not significant.

Root cell wall content was significantly influenced by cultivar, Cd exposure, and Fe supply, as well as by the interactions between cultivar and Fe supply and between Cd exposure and Fe supply (Figure 5A). Overall, Fenghua 1 consistently exhibited higher root cell wall content than Silihong. Fe deficiency reduced cell wall content in Fenghua 1 but had no significant effect in Silihong. Notably, while Cd exposure alone did not significantly alter root cell wall content, a marked increase was observed under combined Cd and Fe deficiency treatment.

Although neither Cd exposure nor Fe deficiency alone significantly affected pectin content in root cell walls, their combination led to a substantial increase in both cultivars (Figure 5B). Hemicellulose 1 (HC1) content decreased under Fe deficiency in Fenghua 1 but remained unchanged in Silihong (Figure 5C). Cd exposure alone reduced HC1 content in both cultivars, with a more pronounced reduction under Fe-deficient conditions. Similarly, Fe deficiency decreased hemicellulose 2 (HC2) content in Fenghua 1, whereas it was unaffected in Silihong (Figure 5D). In contrast, Cd exposure alone increased HC2 content in Silihong but reduced it in Fenghua 1. Cellulose content was significantly elevated under Fe deficiency in both cultivars regardless of Cd exposure, with Cd treatment exerting no additional effect (Figure 5E). Lignin content decreased under Fe deficiency in both cultivars in the absence of Cd; conversely, Cd exposure increased lignin content irrespective of Fe supply status (Figure 5F).

### 2.6. Fe and Cd Content in Root Cell Wall Component

**Figure 6:**
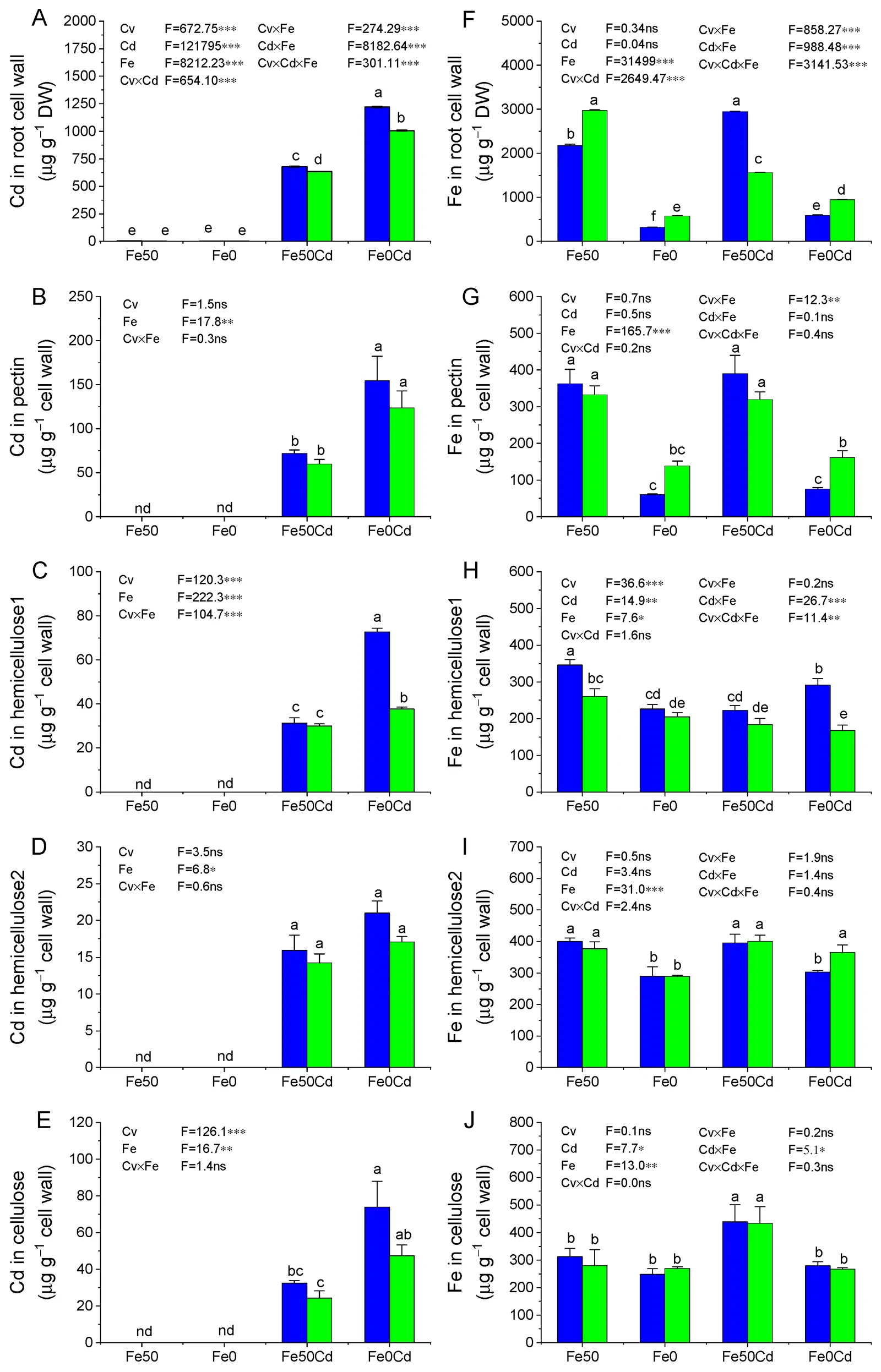
Cadmium and iron in cell wall (**A**,**B**), pectin (**C**,**D**), hemicellulose 1 (**E**,**F**), hemicellulose 2 (**G**,**H**), and cellulose (**I**,**J**) of Fe-sufficient (Fe50) or Fe-deficient (Fe0) plants of Silihong (blue columns) and Fenghua 1 (green columns) exposed to 0 or 2 μM of CdCl_2_ for 14 days. Data (means ± SE, *n* = 3) sharing the same letter(s) above the error bars are not significantly different at the 0.05 level according to Duncan’s multiple range test. Cv, cultivar; * *p* < 0.05, ** *p* < 0.01, and *** *p* < 0.001; ns, not significant; nd, not determined.

The two cultivars differed in their cell wall Cd contents, which were significantly influenced by Fe supply as well as by the interaction between cultivar and Fe supply (Figure 6A). Overall, Silihong exhibited higher Cd contents in cell walls than Fenghua 1. Fe deficiency increased cell wall Cd contents in both cultivars, with a more pronounced increase in Silihong. Compared to Fenghua 1, Silihong accumulated more Cd in the HC1 and cellulose fractions, particularly under Fe-deficient conditions, while Cd contents in pectin and HC2 were similar between the two cultivars (Figure 6B–E). Fe deficiency significantly increased Cd contents in pectin, HC1, and cellulose, but did not affect those in HC2 (Figure 6B–E).

Fe contents in cell walls were higher in Fenghua 1 than in Silihong and were markedly reduced by Fe deficiency (Figure 6F). Cd exposure alone increased cell wall Fe contents in Silihong but decreased them in Fenghua 1 (Figure 6F). Fe contents in pectin were reduced by Fe deficiency but were not affected by Cd exposure (Figure 6G). The two cultivars exhibited similar pectin Fe contents under Fe-sufficient conditions; however, under Fe-deficient conditions, pectin Fe contents were higher in Fenghua 1 than in Silihong (Figure 6G). Compared with Fenghua 1, Silihong had higher Fe contents in HC1, which were reduced by Fe deficiency or Cd exposure alone, as well as by their combination (Figure 6H). Fe contents in HC2 and cellulose were similar between the two cultivars and were reduced by Fe deficiency (Figure 6I,J). Cd exposure increased cellulose Fe contents in both cultivars, while HC2 Fe contents remained unaffected (Figure 6I,J).

### 2.7. Principal Component Analysis (PCA)

**Figure 7:**
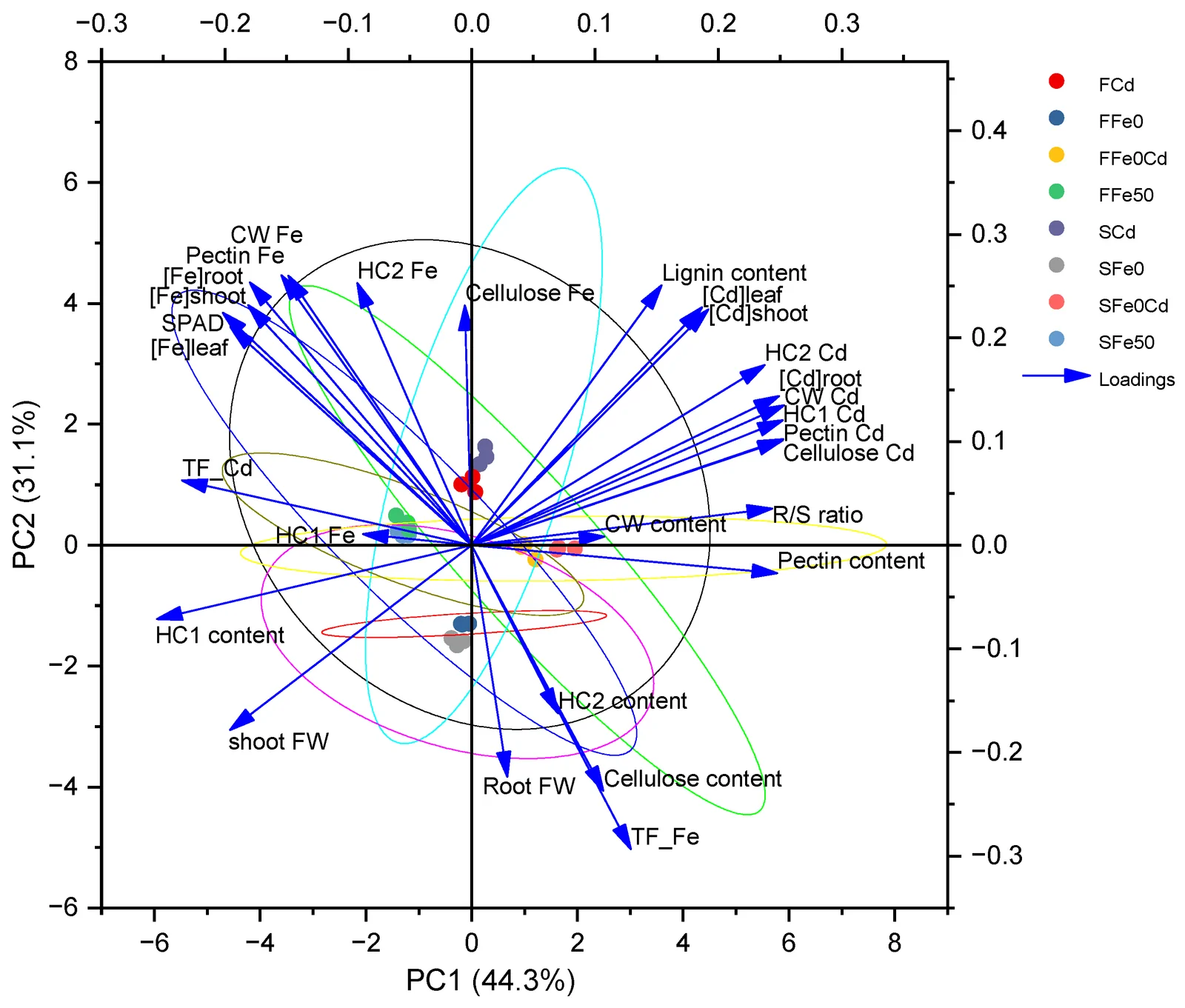
Principal component analysis (PCA) of all variables tested in the peanut cultivars Silihong (S) and Fenghua 1 (F) exposed to Cd (0 or 2 μM) under Fe-sufficient (50 μM) or Fe-deficient (0 μM) conditions.

To further elucidate the effects of Fe/Cd treatments and the contribution of individual variables to the overall variation, a PCA was performed. The first two principal components accounted for 75.4% of the total variance across cultivars and treatments (PC1: 44.3%; PC2: 31.1%), indicating a clear separation among the experimental groups. Figure 7 illustrates the sample distribution in the principal component space, where each point represents an individual sample and colors denote cluster affiliation. The 95% confidence ellipses depict the dispersion of each cluster. Although all eight groups were distinguishable, the two cultivars within each Fe/Cd treatment clustered together. This suggests that the effects of the treatments had a stronger influence on the measured variables than the differences between cultivars.

The variables contributing to the principal components are shown in Figure 7. PC1 was primarily dominated by Cd contents in the cell wall (CW), pectin, HC1, and cellulose; root Cd content; pectin content; root-to-shoot (R/S) ratio; HC2 Cd content; TF_Cd; and HC1 content, with loadings of 0.253, 0.253, 0.252, 0.252, 0.249, 0.248, 0.244, 0.238, −0.235, and −0.255, respectively (Table S1). Among these, root Cd content was positively correlated with Cd contents in the CW, pectin, HC1, and cellulose, but negatively correlated with HC1 content.

PC2 was mainly driven by Fe contents in pectin, CW, roots, HC2, and shoots; lignin content; cellulose Fe content; cellulose content; and TF_Fe, with loadings of 0.261, 0.260, 0.254, 0.253, 0.251, 0.232, 0.231, −0.237, and −0.293, respectively (Table S1). Within this component, CW Fe content was positively correlated with Fe contents in pectin, roots, and shoots, but negatively correlated with cellulose content and TF_Fe (Figure 7).

### 2.8. Hub Gene Identification Using Weighted Gene Co-Expression Network Analysis

To identify key genes regulating cell wall modification under Fe deficiency, Cd exposure, or their combined stress, differentially expressed genes (DEGs) were analyzed using transcriptome data. Pairwise comparisons identified 3535, 1619, 10,233, 8835, and 8275 DEGs for SFe0 vs. SFe50, SFe50Cd vs. SFe50, SFe0Cd vs. SFe50, SFe0Cd vs. SFe0, and SFe0Cd vs. SFe50Cd in Silihong, respectively (Tables S2 and S3). In Fenghua 1, 2274, 1893, 6006, 4974, and 4631 DEGs were detected for FFe0 vs. FFe50, FFe50Cd vs. FFe50, FFe0Cd vs. FFe50, FFe0Cd vs. FFe0, and FFe0Cd vs. FFe50Cd, respectively (Table S2). Cross-cultivar comparisons under identical treatments revealed 6001, 7901, and 8071 DEGs for SFe0 vs. FFe0, SFe50Cd vs. FFe50Cd, and SFe0Cd vs. FFe0Cd, respectively (Tables S2 and S3).

**Figure 8:**
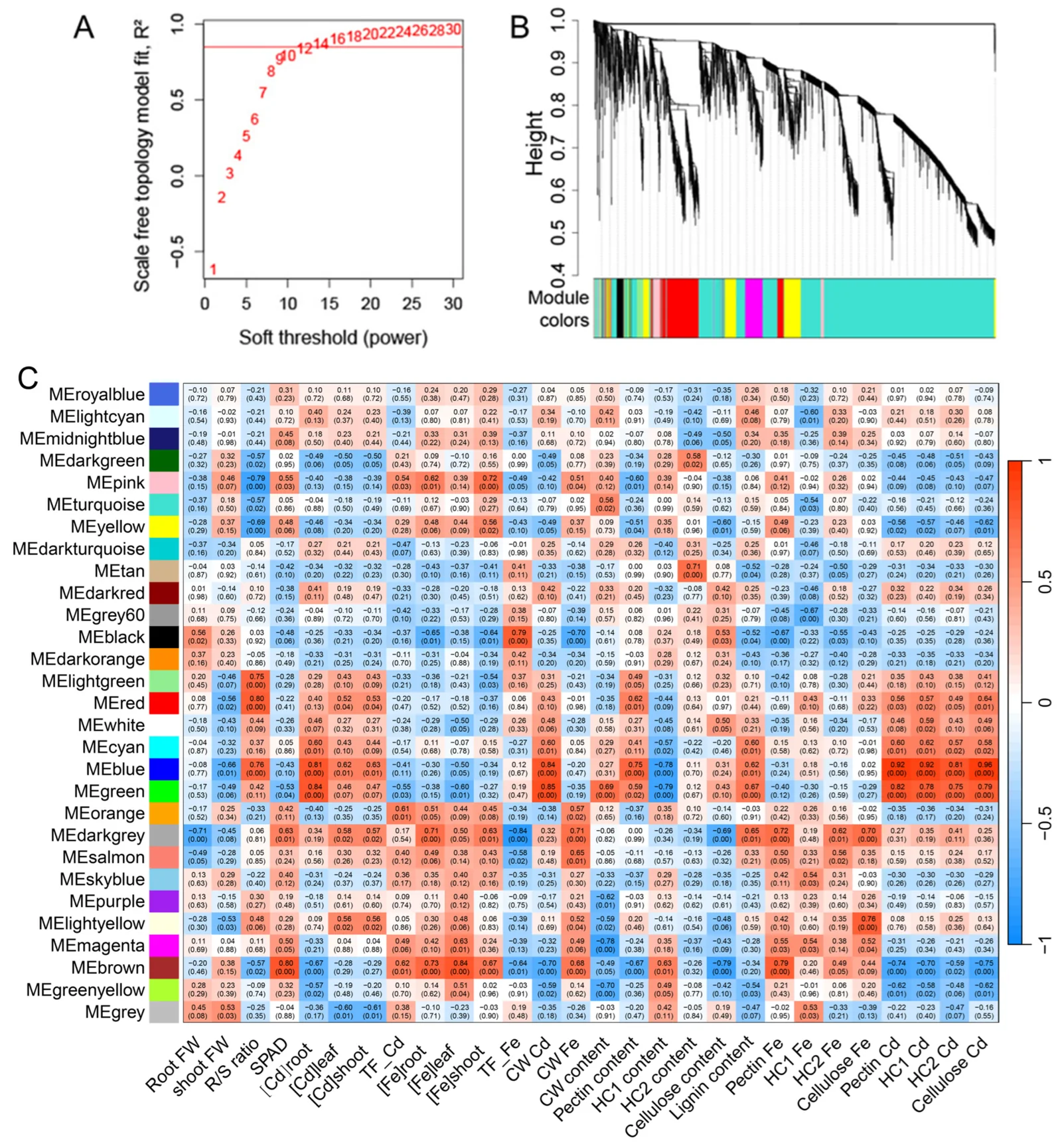
Weighted gene co-expression network analysis (WGCNA) of DEGs in peanut roots exposed to Cd (0 or 2 μM) under Fe-sufficient (50 μM) or Fe-deficient (0 μM) conditions. (**A**) The soft thresholds power (β) based on the scale-free topological fit index R^2^ = 0.90. (**B**) Clustering dendrograms exhibiting genes with similar expression patterns were merged into co-expression modules. (**C**) Correlations between gene modules and phenotype.

To identify distinct co-expressed gene modules highly correlated with the phenotypes or traits described above, a WGCNA was performed using the FPKM values of 20,890 DEGs annotated in the peanut genome. Based on a scale-free topological fit index (R^2^ = 0.90), a soft-thresholding power of β = 14 was selected to construct a hierarchical clustering tree (Figure 8A). The resulting dendrogram clustered the 20,890 DEGs into 29 distinct co-expression modules, designated as royalblue, lightcyan, midnightblue, darkgreen, pink, turquoise, yellow, darkturquoise, tan, darkred, grey60, black, darkorange, lightgreen, red, white, cyan, blue, green, orange, darkgrey, salmon, skyblue, purple, lightyellow, magenta, brown, greenyellow, and grey (Figure 8B). Among these modules, the blue and green modules showed strong positive correlations with pectin and lignin contents in root cell walls, as well as with Cd concentrations in roots, shoots, leaves, and root cell wall fractions (Figure 8C). By contrast, they were negatively correlated with HC1 content in root cell walls. The brown module was positively correlated with SPAD values, HC1 content, root-to-shoot Cd translocation factors, and Fe concentrations in roots, shoots, leaves, root cell walls, and the pectin fraction. Conversely, it exhibited negative correlations with root-to-shoot ratios, pectin and cellulose contents in root cell walls, root-to-shoot Fe translocation factors, and Cd concentrations in roots, root cell walls, and their components (Figure 8C). Thus, the three modules can be considered as the hub modules.

**Figure 9:**
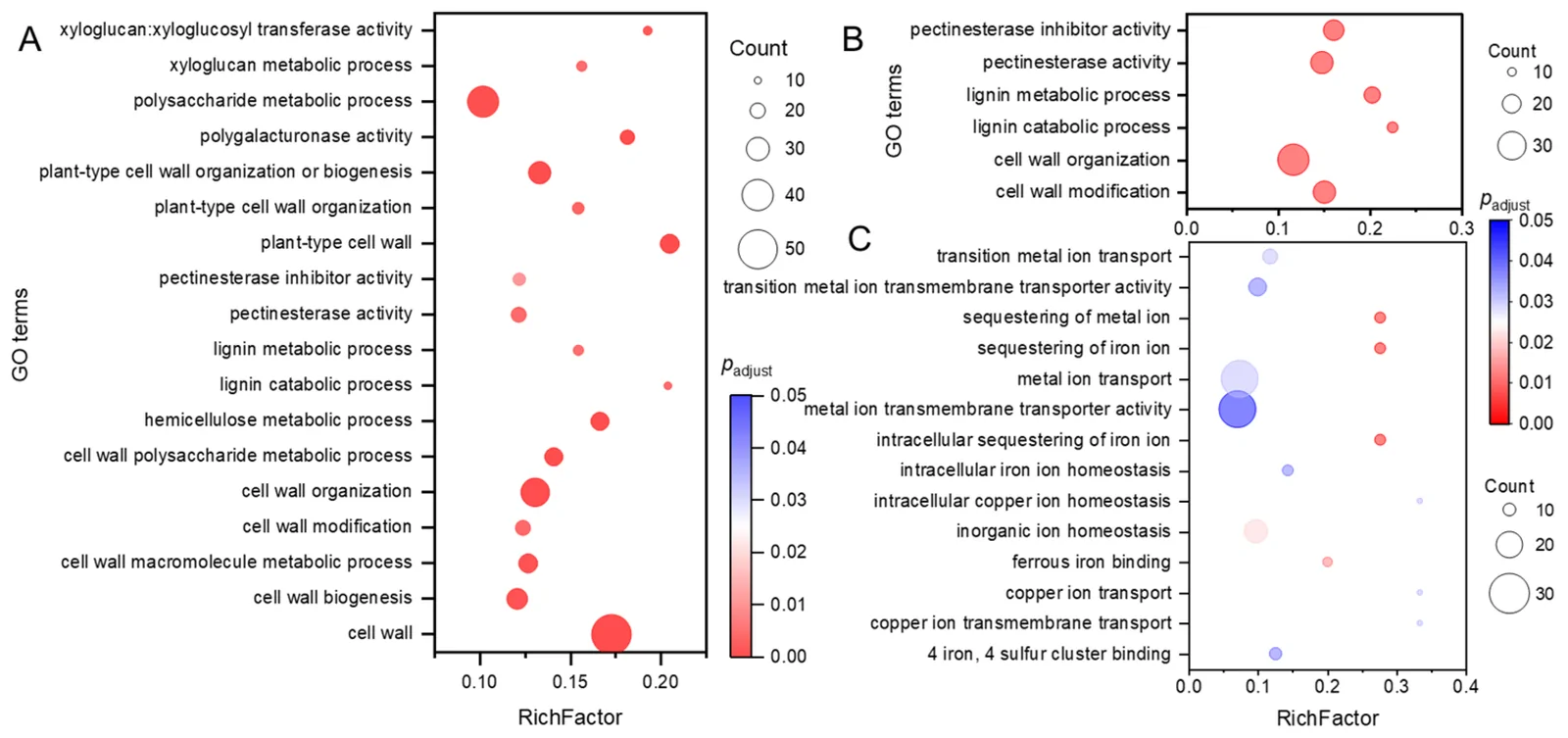
Gene Ontology (GO) enrichment analysis of differentially expressed genes (DEGs) in the three hub modules identified by WGCNA. (**A**) Cell wall-related GO terms significantly enriched in the blue module. (**B**) Cell wall-related GO terms significantly enriched in the brown module. (**C**) Metal transport and sequestration-related GO terms enriched in the green module; no significant enrichment of cell wall-related GO terms was observed in this module.

To elucidate the biological functions of co-expressed DEGs within the three hub modules, Gene Ontology (GO) enrichment analysis was performed. The analysis revealed that DEGs in the brown module were significantly enriched in 171 GO terms (adjusted *p* < 0.05, Table S4). Notably, 18 of these terms were related to cell wall modification, including polysaccharide metabolic process (39 DEGs), cell wall organization (36 DEGs), plant-type cell wall organization or biogenesis (28 DEGs), cell wall macromolecule metabolic process (24 DEGs), hemicellulose metabolic process (23 DEGs), cell wall polysaccharide metabolic process (23 DEGs), cell wall modification (19 DEGs), pectinesterase activity (19 DEGs), polygalacturonase activity (18 DEGs), pectinesterase inhibitor activity (16 DEGs), lignin metabolic process (13 DEGs), cell wall (13 DEGs), xyloglucan metabolic process (13 DEGs), xyloglucan:xyloglucosyl transferase activity (11 DEGs), and lignin catabolic process (10 DEGs) (Figure 9A).

DEGs in the blue module were significantly enriched in 148 GO terms (adjusted *p* < 0.05, Table S5). Among these, six terms were associated with cell wall modification, including cell wall organization (32 DEGs), lignin catabolic process (32 DEGs), cell wall modification (23 DEGs), pectinesterase activity (23 DEGs), pectinesterase inhibitor activity (21 DEGs), and lignin metabolic process (17 DEGs) (Figure 9B).

DEGs in the green module were significantly enriched in 77 GO terms (adjusted *p* < 0.05, Table S6). The majority of these terms were associated with metal ion transport, sequestration, and tolerance, including metal ion transmembrane transporter activity (27 DEGs), metal ion transport (27 DEGs), inorganic ion homeostasis (17 DEGs), transition metal ion transmembrane transporter activity (13 DEGs), transition metal ion transport (11 DEGs), 4 iron, 4 sulfur cluster binding (9 DEGs), sequestering of metal ion (8 DEGs), sequestering of iron ion (8 DEGs), intracellular iron ion homeostasis (8 DEGs), intracellular sequestering of iron ion (8 DEGs), and ferrous iron binding (8 DEGs) (Figure 9C). No significantly enriched terms related to cell wall modification were identified in this module.

**Figure 10:**
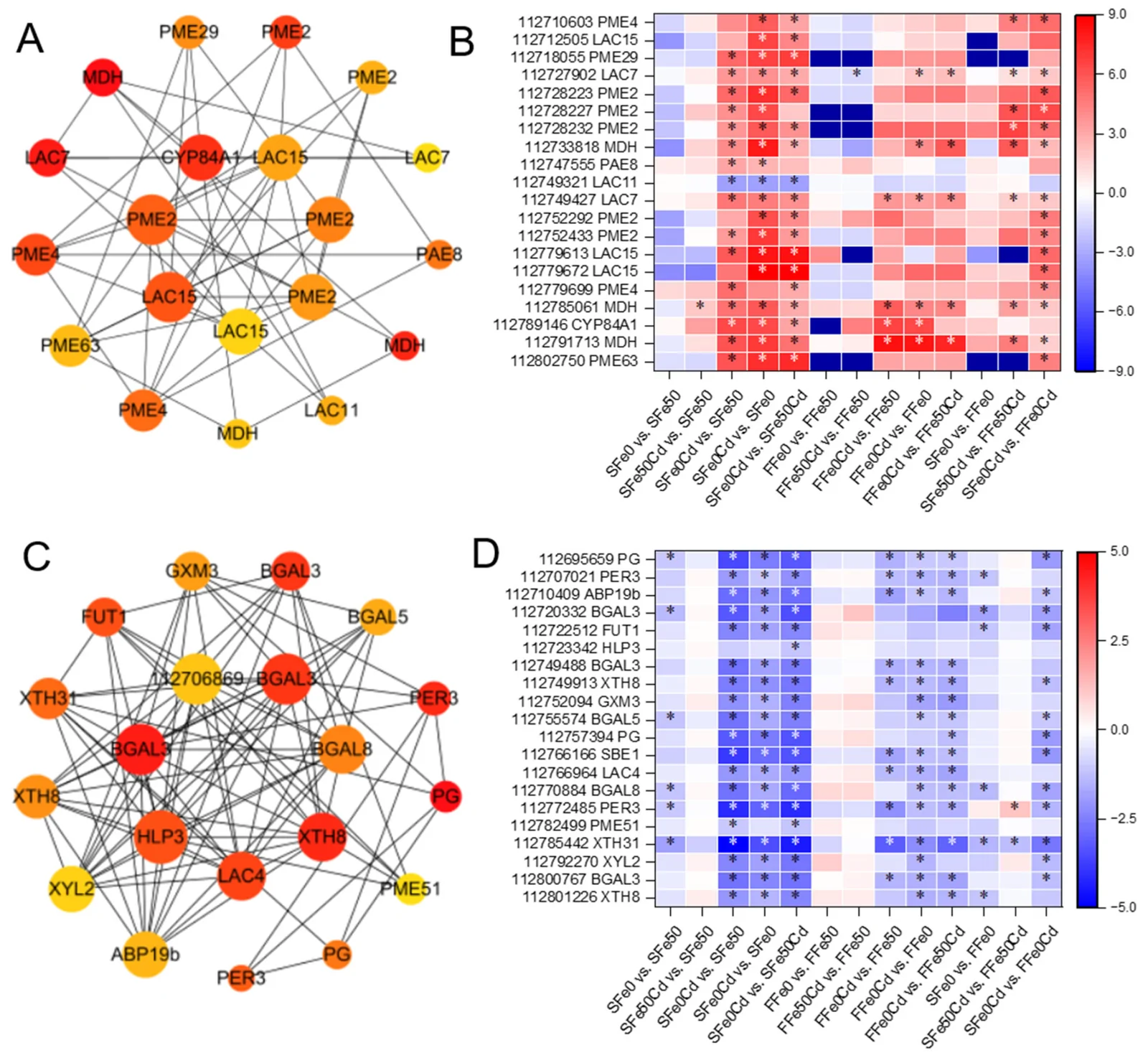
Co-expression networks and expression profiles of the top 20 hub genes in the blue and brown modules identified using the MCC method. (**A**) Hub gene co-expression network and (**B**) log_2_ (fold change) of hub genes in the blue module. (**C**) Hub gene co-expression network and (**D**) log_2_ (fold change) of hub genes in the brown module. Asterisks (*) indicate genes that were differentially expressed in a specific comparison.

To identify hub genes involved in cell wall modification within the blue and brown modules, eigengenes were selected using stringent weight thresholds (≥0.2 for the blue module and ≥0.15 for the brown module). After removing genes unrelated to cell wall modification, co-expression networks were constructed using Cytoscape. Based on these networks, the top 20 hub genes were identified using the MCC algorithm from the cytoHubba plugin (Figure 10). As shown in Figure 10A, the top 20 hub genes in the blue module comprised nine pectinesterases (five *PME2*, two *PME4*, one *PME29*, and one *PME63*), six laccases (three *LAC15*, two *LAC7*, and one *LAC11*), three mannitol dehydrogenases (*MHD*s), and one cytochrome P450 84A1 (*CYP84A1*). The majority of these hub genes were induced specifically by the combined Cd and Fe deficiency treatment, while remaining unchanged under Cd exposure or Fe deficiency alone (Figure 10B). The induction was more pronounced in Silihong than in Fenghua 1. As a result, Silihong exhibited higher expression levels of these hub genes than Fenghua 1 under Cd exposure, particularly under the combined treatment.

In the brown module, the top 20 hub genes included five beta-galactosidases (three *BGAL3*, one *BGAL5*, and one *BGAL8*), three mannitol dehydrogenases (*MHD*s), three xyloglucan endotransglucosylase/hydrolase proteins (two *XTH8* and one *XTH31*), two probable polygalacturonases (*PG*s), as well as beta-xylosidase/alpha-L-arabinofuranosidase 2 (*XYL2*), galactoside 2-alpha-L-fucosyltransferase (*FUT1*), glucuronoxylan 4-O-methyltransferase 3 (*GXM3*), heparanase-like protein 3 (*HLP3*), auxin-binding protein ABP19b (*ABP19b*), and *LAC4* (Figure 10C). Almost all of these hub genes were suppressed by the combined Cd and Fe deficiency treatment, an effect that was more pronounced in Silihong than in Fenghua 1 (Figure 10D). Specifically, Fe deficiency repressed the expression of *PG*, *BGAL3*, *BGAL5*, *BGAL8*, *PER3*, and *XTH31* in Silihong. As a result, under Fe-deficient conditions, Silihong exhibited lower expression levels of these hub genes than did Fenghua 1.

### 2.9. qRT-PCR Validation of Hub Genes

**Figure 11:**
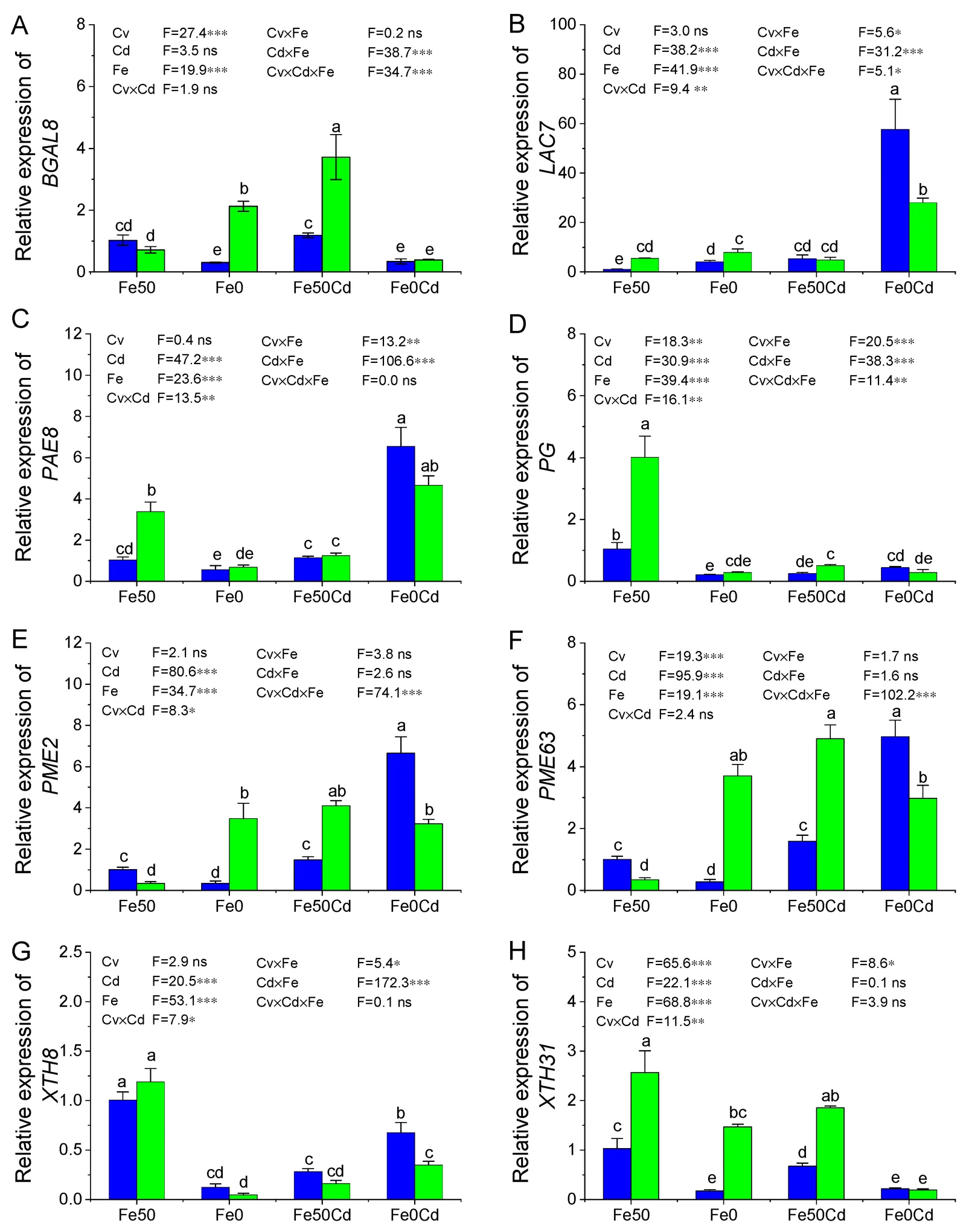
Relative expression of hub genes in the roots of Fe-sufficient (Fe50) or Fe-deficient (Fe0) plants of Silihong (blue columns) and Fenghua 1 (green columns) exposed to 0 or 2 μM of CdCl_2_ for 14 days. (**A**) *BGAL8*; (**B**) *LAC7*; (**C**) *PAE8*; (**D**) *PG*; (**E**) *PME2*; (**F**) *PME63*; (**G**) *XTH8*; (**H**) *XTH31*. Data (means ± SE, *n* = 3) sharing the same letter(s) above the error bars are not significantly different at the 0.05 level according to Duncan’s multiple range test. Cv, cultivar; * *p* < 0.05, ** *p* < 0.01, and *** *p* < 0.001; ns, not significant.

To validate the transcriptome data, eight hub genes involved in cell wall modification were selected for qRT-PCR analysis. As shown in Figure 11, Fe deficiency down-regulated the expression of *PAE8*, *PG*, *XTH8*, and *XTH31* in both cultivars, whereas the remaining four genes exhibited a cultivar-dependent response to Fe deficiency. Cadmium exposure alone repressed *PG* and *XTH8* expression in both cultivars, while the other genes showed cultivar-dependent patterns (Figure 11). Combined Fe deficiency and Cd exposure repressed the expression of *BGAL8*, *PG*, *XTH8*, and *XTH31*, but induced the expression of *LAC7*, *PAE8*, *PME2*, and *PME63* in both cultivars (Figure 11). The qRT-PCR data, consistent with the WGCNA results, indicate that these genes play important roles in cell wall modification under combined Fe deficiency and Cd exposure.

## 3. Discussion

### 3.1. Cultivar-Specific Fe Deficiency Responses and Cd Accumulation in Peanut

The two cultivars exhibited distinct responses to Fe deficiency. Silihong increased root fresh weight under Fe deficiency (Figure 1A), reflecting enhanced root exploration for Fe acquisition, which is a typical Strategy I response [41]. Fenghua 1 lacked this response, confirming its sensitivity to Fe deficiency. Cd exposure reduced shoot growth in both cultivars, an effect exacerbated by combined Fe deficiency and Cd stress (Figure 1B). Despite this overall reduction, Fenghua 1 maintained relatively better growth under Cd stress, suggesting a higher capacity for Cd tolerance.

Consistent with previous reports in peanut [38,39,40,42] and other plant species [7,9,10,11,12], Fe deficiency significantly increased root Cd concentrations in both cultivars (Figure 2A). However, Cd translocation to shoots was markedly reduced under Fe deficiency, particularly in the Fe deficiency-tolerant cultivar Silihong, which exhibited higher root Cd accumulation but lower shoot Cd concentrations (Figure 2D). This paradoxical response, enhanced uptake coupled with restricted translocation, is consistent with findings in pea [11] and rice [9] and points to a root-based retention mechanism operating downstream of initial Cd uptake.

### 3.2. Cell Wall Remodeling Enhances Cd Retention in Peanut Roots

The interaction between Fe and Cd accumulation in plants is complex. In some species, such as *Arabidopsis*, barley, and rice, excess Fe inhibits Cd accumulation in roots and shoots [43,44,45]. In others, including hybrid poplar (*Populus tremula* × *P. alba*) and *P. cathayana*, Fe supply enhances Cd accumulation in both roots and shoots [46,47]. These contrasting observations, together with our findings, suggest that additional mechanisms, beyond simple competition for shared transporters, underlie the complex interaction between Fe and Cd in plants.

Our results clearly revealed an antagonistic relationship between Cd and Fe in peanut plants. Root Cd concentrations were negatively correlated with Fe concentrations in roots, shoots, and leaves, yet positively correlated with root-to-shoot Fe translocation factors. A negative correlation was also observed between the translocation factors of Cd and Fe, further supporting an antagonistic interaction between the transport pathways of these two metals. Moreover, the positive correlation between root Cd concentration and Cd content in the cell wall highlights the crucial role of cell walls in root Cd retention.

Cell walls, the outermost structures of plant cells, not only serve as protective barriers for the protoplast but also function as effective biosorbents for Cd, thereby alleviating the toxicity of this heavy metal [48]. Because cell walls are rich in functional groups such as –COOH, –OH, –NH_2_ and –SH, they can bind metal cations, making them a sink for trace metals, including Cd and Fe [49,50,51]. Importantly, cell wall composition is dynamic and can be modulated by nutritional stress, thereby directly influencing its Fe- and Cd-binding capacity.

Our findings demonstrate that Fe deficiency and Cd exposure significantly alter the content and composition of root cell walls (Figure 5). Pectin, a complex polysaccharide comprising homogalacturonan, rhamnogalacturonan I, rhamnogalacturonan II, and xylogalacturonan, provides negatively charged carboxyl groups for cation binding, primarily through its homogalacturonan domains [50]. Notably, pectin content increased only under combined Fe deficiency and Cd exposure (Figure 5B). This increase was positively correlated with root Cd concentrations, Cd contents in root cell walls and the pectin fraction, and root-to-shoot Fe translocation factors, while it was negatively correlated with Fe concentrations in roots, shoots, and leaves. Collectively, these findings suggest that the enhanced pectin content under combined stress promotes Cd immobilization in the root apoplast.

As a complex heterogeneous polysaccharide composed of xyloglucans, xylans, and glucomannans, hemicellulose acts as a major site for metal ion sequestration in root cell walls [21,22]. In the present study, we found that although Cd exposure reduced HC1 content, an effect further intensified under Fe-deficient conditions (Figure 5C), Cd accumulation in this fraction was increased (Figure 6C). Correlation analysis further revealed that HC1 content was negatively correlated with Cd concentrations in roots, Cd contents in root cell walls and the HC1 fraction, while it was positively correlated with Fe concentrations in plant tissues and Fe contents in root cell walls and the HC1 fraction. It has been reported that decreased hemicellulose content in cell walls facilitates the reutilization of apoplastic Fe in roots, thereby alleviating Fe deficiency [34,52]. Therefore, competition between Fe and Cd for binding sites in the HC1 fraction contributes to the Fe deficiency-induced increase in Cd retention in roots and the consequent reduction in Cd accumulation in shoots.

Cellulose, a linear polysaccharide composed of glucose residues linked by β-1,4-glycosidic bonds, contains various functional groups, including carbonyl, phenolic, and amino groups, that provide abundant binding and adsorption sites for metal ions, thereby contributing to heavy metal stress tolerance [53]. Despite limited research on the direct binding of Cd to cellulose, a previous study reported a significant increase in cellulose Cd content upon Cd exposure [54]. In the present study, cellulose content increased under Fe deficiency in both cultivars (Figure 5E), accompanied by a corresponding increase in Cd accumulation in this fraction (Figure 6E). Correlation analysis further revealed that cellulose content was positively correlated with Cd concentrations in roots, Cd contents in root cell walls and the cellulose fraction, while it was negatively correlated with Fe concentrations in plant tissues and Fe contents in root cell walls and the cellulose fraction. Together, these results indicate that enhanced cellulose content under Fe deficiency promotes Cd immobilization in the root apoplast.

Lignin is a complex aromatic biopolymer that acts as a key physical barrier, preventing metal ions from entering cells by reducing penetration through enhanced cell wall lignification. Our results show that lignin content decreased under Fe deficiency but increased upon Cd exposure (Figure 5F). The reduction in lignin under Fe-deficient conditions may represent an adaptive strategy to facilitate Fe translocation from roots to shoots, as a less rigid cell wall could enhance Fe mobility. Conversely, the Cd-induced increase in lignin content likely reinforces the physical barrier, reducing Cd penetration and subsequent translocation, consistent with its established role in metal detoxification. These opposing lignin responses to Fe and Cd stress highlight the dynamic and stress-specific remodeling of cell wall components.

Taken together, Fe deficiency enhances Cd sequestration in peanut root cell walls by modulating their composition. The increase in pectin content and its associated Cd accumulation plays a crucial role in this process. In contrast, the reduction in HC1 content under Fe deficiency may increase the mobilization and root-to-shoot translocation of Fe, thereby enhancing the competitive capacity of Cd for metal-binding sites in the cell wall.

### 3.3. Hub Genes Governing Cell Wall Modification

In previous studies, we performed comparative transcriptome analyses to identify key genes involved in Fe and/or Cd transport in peanut roots [55,56]. Here, based on these transcriptome data, we applied the WGCNA approach to identify hub genes associated with cell wall modification. The results revealed that three hub modules (blue, brown, and green) were strongly associated with phenotypic traits (Figure 8). The blue module, positively correlated with pectin and lignin contents as well as Cd accumulation in cell wall fractions, contained hub genes predominantly encoding pectinesterases (*PME*s) and laccases (*LAC*s) (Figure 10A). In contrast, the brown module, positively correlated with HC1 content and Fe accumulation but negatively correlated with Cd accumulation, contained hub genes encoding beta-galactosidases (*BGAL*s), xyloglucan endotransglucosylase/hydrolases (*XTH*s), and polygalacturonases (*PG*s) (Figure 10C). These genes are involved in the degradation and modification of pectin and hemicellulose. The green module, enriched in metal ion transport-related GO terms, likely represents the well-characterized Fe deficiency-induced uptake system, including *NRAMP1* transporters. The absence of cell wall-related terms in this module confirms that the cell wall modification machinery operates independently of, but coordinately with, the metal uptake system. Together, these results provide a comprehensive view of the molecular networks underlying Fe–Cd interactions.

Pectin modification is mediated by several classes of enzymes, including pectin methylesterase (PME), polygalacturonase (PG), and pectin acetylesterase (PAE) [57]. PMEs catalyze the demethylesterification of pectin, exposing negatively charged carboxyl groups that enhance Cd binding capacity [58]. The specific induction of multiple *PME* genes (*PME2*, *PME4*, *PME29*, and *PME63*) under combined Fe deficiency and Cd treatment (Figure 10B and Figure 11) suggests that PME-mediated pectin demethylesterification might play a crucial role in Cd sequestration and detoxification in peanut roots.

In addition to methylesterification, acetylation is another important modification of pectins. This modification is mediated by pectin acetylesterases (PAEs), which cleave acetyl ester bonds from the pectin backbone. Loss-of-function mutants of PAE-encoding genes, such as *pae8* and *pae9*, have been shown to exhibit reduced inflorescence stem growth [59]. In the present study, the up-regulation of *PAE8* under combined Fe deficiency and Cd treatment in Silihong (Figure 10B and Figure 11) underscores the potential role of PAE-mediated deacetylation in the response to Cd stress.

PGs are major pectin-hydrolyzing enzymes that catalyze the cleavage of α-1,4-glycosidic linkages between galacturonic acid residues in homogalacturonan, leading to cell wall loosening and cell separation [60]. This enzymatic activity may consequently affect the metal-binding capacity of the cell wall by altering pectin integrity. For instance, overexpression of *OsPG2* in rice decreased pectin content in both leaves and roots, while enhancing aluminum accumulation in the root elongation zone [61,62]. In the present study, the suppression of *PG* genes under combined Fe deficiency and Cd exposure (Figure 10D and Figure 11) may inhibit pectin hydrolysis, thereby contributing to Cd retention. This is consistent with the increased pectin content and its Cd accumulation observed under combined stress (Figure 5B and Figure 6B).

BGALs are glycosyl hydrolases that remove β-D-galactosyl residues from the non-reducing termini of various polysaccharides, including galactans, arabinogalactans, arabinogalactan proteins, and xyloglucans, thereby contributing to cell wall remodeling in diverse plant developmental processes and stress responses [63]. Notably, Cd and Hg exposure have been shown to strongly inhibit BGAL activity [64]. Similarly, our results showed that combined Fe deficiency and Cd exposure significantly repressed the expression of *BGAL3*, *BGAL5*, and *BGAL8* in peanuts, particularly in Silihong (Figure 10D and Figure 11). This inhibition of BGALs may contribute to pectin accumulation, thereby enhancing Cd retention capacity.

XTHs exhibit xyloglucan endotransglucosylase (XET) and/or endohydrolase (XEH) activities, mediating the cleavage and reconnection of xyloglucan crosslinks in the cell wall, and have been implicated in heavy metal detoxification [65,66]. For instance, knockout of *XTH31* in *Arabidopsis* increases Al resistance by reducing xyloglucan accumulation in hemicellulose, thereby decreasing Al retention in the cell wall [66]. Similarly, *XTH18* is upregulated by Pb in *Arabidopsis* root tips, and the *xth18* mutant exhibits reduced Pb sensitivity [67]. Overexpression of *P. euphratica PeXTH* in tobacco enhances Cd tolerance by increasing xyloglucan degradation, which reduced cell wall Cd^2+^ binding sites and restricts root Cd uptake [65]. In the present study, the suppression of *XTH8* and *XTH31* under combined Fe deficiency and Cd exposure (Figure 10D and Figure 11), particularly in Silihong, may decrease xyloglucan accumulation in root cell wall hemicellulose, thereby facilitating Fe translocation to shoots.

Plant laccases are phenol-oxidizing metalloenzymes produced extracellularly that primarily participate in lignin polymerization and have been implicated in heavy metal tolerance [68]. The induction of *LAC* genes under combined stress likely contributes to lignin deposition and Cd immobilization, as evidenced by increased lignin content and reduced root-to-shoot Cd translocation.

**Figure 12:**
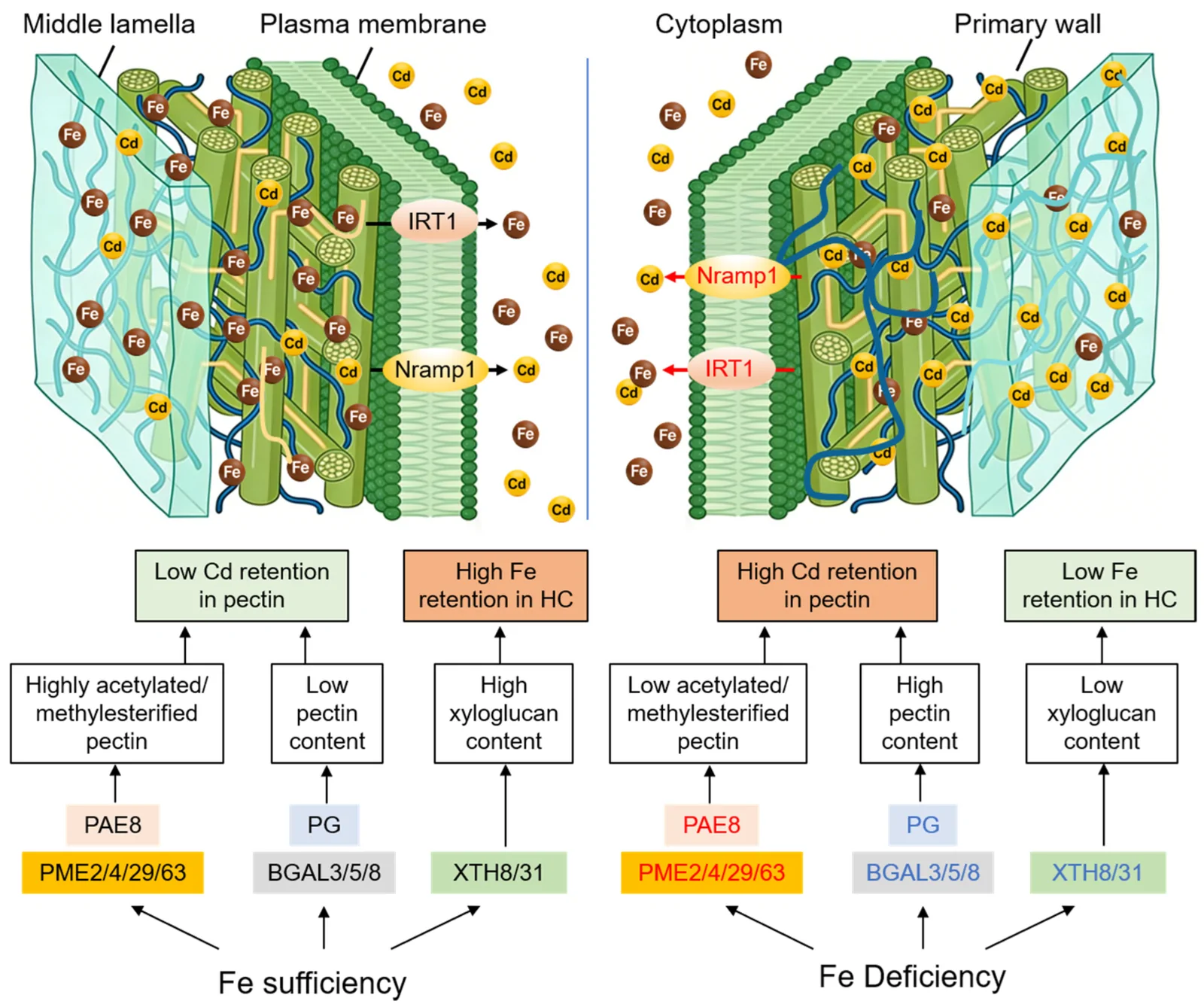
Schematic representation of cell wall modifications regulating iron (Fe) and cadmium (Cd) retention and bioavailability in roots. Pectin (blue lines) and hemicellulose (HC, yellow lines) in the root cell wall act as major reservoirs for competitive Fe/Cd binding. Highly acetylated/methylesterified pectin may reduce Cd retention and enhance Cd mobility, whereas demethylesterified pectin may increase Cd immobilization. The abundance of cell wall polymers may also modulate Cd retention in the apoplast. Enzymes highlighted in red and blue indicate genes that were up- or down-regulated, respectively, by combined Cd exposure and iron deficiency.

Based on these findings, we propose a conceptual model for how Fe deficiency restricts Cd translocation in peanut (Figure 12). Under Fe-sufficient conditions, Cd entering the root is partially bound by cell wall components, but a substantial portion is loaded into the xylem and transported to shoots. Under Fe deficiency, two parallel processes operate: (1) upregulation of Fe uptake transporters (e.g., *IRT1* and *NRAMP1*) increases Cd influx into root cells; (2) structural remodeling of the cell wall might enhance the apoplastic Cd reservoir. Specifically, induction of *PAE8* and *PME*s under combined Fe deficiency and Cd exposure might increase pectin content, deacetylation, and demethylesterification, while repression of *PG*s and *BGAL*s reduces pectin hydrolysis, thereby generating more negative binding sites for Cd. Concurrently, suppression of *XTH*s may alter hemicellulose composition, facilitating Fe mobilization and translocation to shoots.

The cultivar Silihong exhibits more effective cell wall remodeling under combined stress, which may be characterized by higher expression of *PME* and *LAC* genes and stronger suppression of *PG*s, *BGAL*s, and *XTH*s, explaining its superior ability to retain Cd in roots under Fe deficiency. Although this study establishes a key role for cell wall remodeling in Cd retention under Fe-deficient conditions, the functional roles of these cell wall modification genes have not been experimentally validated. Moreover, determination of the degree of pectin methylesterification would have further bolstered the mechanistic model. Therefore, the underlying molecular mechanisms and the precise nature of Cd binding sites warrant further investigation.

## 4. Materials and Methods

### 4.1. Plant Culture and Treatment

Two peanut cultivars differing in their tolerance to iron deficiency, Fenghua 1 (Fe-deficiency-sensitive) and Silihong (Fe-deficiency-tolerant) [55], were cultivated hydroponically. Seeds were surface-sterilized in 5% sodium hypochlorite, rinsed with deionized water for 24 h at room temperature, and germinated in sterile sand. Three-day-old uniform seedlings were transferred to polyethylene pots containing 3.5 L of nutrient solution as previously described [38]. At ten days old, the seedlings were exposed to Cd stress (0 or 2 μM of CdCl_2_) under either Fe-sufficient (50 μM of Fe-EDTA) or Fe-deficient (0 μM of Fe-EDTA) conditions. The nutrient solution was renewed twice weekly. The experiment was conducted using a factorial completely randomized design, with three biological replicates per treatment/cultivar and four seedlings per biological replicate.

Plants were grown in a controlled-environment growth chamber under a 14-h photoperiod with an average irradiance of 600 μmol m^−2^ s^−1^, day/night temperatures of 28/25 °C, and relative humidity maintained at 54–66%. Pots were randomly rearranged daily to minimize positional effects. After 14 days of treatment, plants were harvested for subsequent analyses.

### 4.2. SPAD and Plant Growth Determination

At the end of the experimental period, fully expanded leaves were selected for chlorophyll content measurement using a SPAD-502Plus meter (Konica Minolta, Inc., Tokyo, Japan). At harvest, plants were separated into roots and shoots, and the fresh weight of each part was recorded.

### 4.3. Analysis of Cd and Fe in Plants

At harvest, the entire root systems were rinsed with 20 mM of Na_2_EDTA for 15 min to remove surface-bound metal ions. The root, leaf, and shoot (stem and leaf) tissues were oven-dried at 70 °C to constant weight. Dried samples were ground into a fine powder and digested in a mixture of HNO_3_ and HClO_4_ (3:1, *v*/*v*) at 180 °C until complete dissolution. Cd and Fe concentrations were determined using an Inductively Coupled Plasma Optical Emission Spectrometer (ICP-OES; Agilent, Santa Clara, CA, USA). The root-to-shoot translocation factor for Cd and Fe was calculated as the ratio of shoot metal concentration to root metal concentration.

### 4.4. Cell Wall Fraction Preparation and Polysaccharide Content Measurement

Cell walls were isolated following the method of Zhong and Läuchli [69]. Briefly, root tissues were ground to a fine powder in liquid nitrogen using a mortar and pestle, then homogenized in 75% ethanol for 20 min in an ice-cold water bath. The homogenate was centrifuged at 8000 rpm for 10 min, and the supernatant was discarded. The pellet was subsequently washed by sequential homogenization and centrifugation in acetone, a 1:1 (*v*/*v*) mixture of methanol and chloroform, and finally methanol. Each washing step lasted 20 min, and the supernatant was removed after each centrifugation. The resulting pellet, comprising the cell wall material, was freeze-dried overnight and stored at 4 °C until further analysis.

Cell wall fractions, namely pectin, HC1, HC2, and cellulose, were isolated from the crude cell wall material following the method of Yang et al. [70]. Pectin was extracted twice with 0.5% ammonium oxalate buffer (containing 0.1% NaBH_4_, pH 4.0) in a boiling water bath for 1 h each, and the supernatants were pooled. The remaining pellet was extracted with 4% KOH (containing 0.1% NaBH_4_) for 24 h, and the supernatants were collected as the HC1 fraction. The resulting pellet was further extracted with 24% KOH (containing 0.1% NaBH_4_) for 24 h, and the pooled supernatants were retained as the HC2 fraction. The remaining pellet was washed twice with ddH_2_O and then incubated with 1 mL of 72% H_2_SO_4_ at 25 °C for 1 h with continuous shaking to solubilize cellulose. Following incubation, 2 mL of ddH_2_O was added, and the mixture was centrifuged at 10,000× *g* for 10 min. The resulting supernatant was retained as the cellulose fraction.

Galacturonic acid, D-xylose, and glucose were used as calibration standards for the quantification of total pectin, hemicellulose (HC1 and HC2), and cellulose fractions, respectively. Lignin content was determined following the method described by Ma et al. [71].

### 4.5. Determination of Cd and Fe in Cell Wall Fractions

Dried cell wall material (0.01 g) was directly digested in a mixture of HNO_3_ and HClO_4_ (3:1, *v*/*v*) at 180 °C. The pectin, HC1, HC2, and cellulose fractions, after evaporation to dryness, were likewise digested using the same acid mixture. Cd and Fe concentrations in all digests were determined using an ICP-OES (Agilent, Santa Clara, CA, USA).

### 4.6. Identification of Differentially Expressed Genes from RNA-Seq Data

Transcriptome profiles of roots from Fenghua 1 and Silihong under different Fe and Cd treatments were obtained from our previously published RNA-seq data [55,56]. DEGs were identified using the DESeq2 R package (v. 1.16.1). Genes with an absolute log_2_ fold change ≥ 1 (corresponding to at least a 2-fold change) and an adjusted *p*-value (Benjamini–Hochberg correction) < 0.05 were considered significantly differentially expressed.

### 4.7. Weighted Gene Co-Expression Network Analysis

A weighted gene co-expression network was constructed using the WGCNA R package (v. 4.4.3). Pearson correlation coefficients were calculated for all DEGs, and the soft-thresholding power β was selected using the pickSoftThreshold function. The correlation matrix was then raised to the selected power β to generate an adjacency matrix, which was subsequently transformed into a topological overlap matrix (TOM) for module detection. Module eigengenes (MEs) were calculated for each identified module, and Pearson correlations between MEs and phenotypic traits were computed to identify trait-associated modules. For the key modules, cell wall-related genes were screened and imported into Cytoscape (v. 3.10.3) for visualization of co-expression networks.

### 4.8. qRT-PCR Analysis

At the end of the experiment, fresh root tissues were collected, immediately snap-frozen in liquid nitrogen, and stored at −80 °C for subsequent qRT-PCR analysis. RNA extraction, cDNA synthesis, and quantitative real-time PCR were performed as previously described in our earlier studies [39,71]. Transcript levels were normalized against the peanut 60S ribosomal protein L7-2 (112697914), which served as the endogenous reference. Primer pairs for the eight target genes and *60S* are provided in Table S7. The relative expression of each gene was determined via the 2^−ΔΔCT^ method [72], using three independent biological replicates per treatment, with three technical replicates per biological sample to ensure reproducibility.

### 4.9. Statistical Analysis

Data were analyzed by one- and two-way analysis of variance (ANOVA) using IBM SPSS Statistics version 22 (IBM, New York, NY, USA). Significant differences among treatment means were assessed by Duncan’s multiple range test at a probability level of 5%. Pearson’s correlation analysis and principal component analysis (PCA) were performed using Origin 2025b (OriginLab Corporation, Northampton, MA, USA).

## 5. Conclusions

This study demonstrates that iron deficiency restricts Cd translocation to peanut shoots by remodeling root cell wall composition and enhancing Cd sequestration capacity. Our findings reveal that Fe deficiency triggers cultivar-specific modifications in cell wall polysaccharides, thereby altering binding sites for Fe and Cd competition. Increased pectin content under combined stress enhances Cd sequestration, while reduced hemicellulose (HC1) content facilitates Fe mobilization to shoots. The Fe-deficiency-tolerant cultivar Silihong exhibited superior Cd immobilization, which may be characterized by higher Cd accumulation in HC1 and cellulose fractions, more pronounced induction of *PME*, *PAE8* and *LAC* genes, and stronger suppression of *XTH*s, *PG*s, and *BGAL*s under combined stress. These findings uncover a structural detoxification mechanism operating downstream of uptake transporters and highlight key cell wall components and regulatory genes as potential targets for breeding peanuts with improved food safety and nutritional quality.

## Data Availability

The original contributions presented in this study are included in the article. Further inquiries can be directed to the corresponding author.

## Supplementary Materials

The following supporting information can be downloaded at: https://www.mdpi.com/article/10.3390/plants15172641/s1, Table S1: Loadings of traits for the top three components; Table S2: Differential expression analysis of the transcriptome for the SFe0 vs. SFe50, SFe50Cd vs. SFe50, SFe0Cd vs. SFe50, SFe0Cd vs. SFe0, SFe0Cd vs. SFe50Cd, FFe0 vs. FFe50, FFe50Cd vs. FFe50, FFe0Cd vs. FFe50, FFe0Cd vs. FFe0, FFe0Cd vs. FFe50Cd, SFe0 vs. FFe0, SFe50Cd vs. FFe50Cd, and SFe0Cd vs. FFe0Cd comparisons; Table S3: DEG distribution in the 13 comparisons shown in Table S2; Table S4: Gene Ontology (GO) enrichment analysis of differentially expressed genes (DEGs) in the brown module identified by WGCNA; Table S5: Gene Ontology (GO) enrichment analysis of differentially expressed genes (DEGs) in the blue module identified by WGCNA; Table S6: Gene Ontology (GO) enrichment analysis of differentially expressed genes (DEGs) in the green module identified by WGCNA; Table S7: Primers of the eight hub genes selected for qRT-PCR analysis.

## References

[1] Haider F.U., Liqun C., Coulter J.A., Cheema S.A., Wu J., Zhang R., Wenjun M., Farooq M. (2021). Cadmium Toxicity in Plants: Impacts and Remediation Strategies. Ecotoxicol. Environ. Saf..

[2] Sterckeman T., Thomine S. (2020). Mechanisms of Cadmium Accumulation in Plants. Crit. Rev. Plant Sci..

[3] Riaz N., Guerinot M.L. (2021). All Together Now: Regulation of the Iron Deficiency Response. J. Exp. Bot..

[4] Merry R., Dobbels A.A., Sadok W., Naeve S., Stupar R.M., Lorenz A.J. (2022). Iron Deficiency in Soybean. Crop Sci..

[5] Pasricha S.-R., Tye-Din J., Muckenthaler M.U., Swinkels D.W. (2021). Iron Deficiency. Lancet.

[6] Zhang Q., Wen Q., Ma T., Zhu Q., Huang D., Zhu H., Xu C., Chen H. (2023). Cadmium-Induced Iron Deficiency Is a Compromise Strategy to Reduce Cd Uptake in Rice. Environ. Exp. Bot..

[7] Bao T., Sun T.H., Sun L.N. (2012). Effect of Cadmium on Physiological Responses of Wheat and Corn to Iron Deficiency. J. Plant Nutr..

[8] Gao C., Wang Y., Xiao D.-S., Qiu C.-P., Han D.-G., Zhang X.-Z., Wu T., Han Z.-H. (2011). Comparison of Cadmium-Induced Iron-Deficiency Responses and Genuine Iron-Deficiency Responses in *Malus xiaojinensis*. Plant Sci..

[9] Nakanishi H., Ogawa I., Ishimaru Y., Mori S., Nishizawa N.K. (2006). Iron Deficiency Enhances Cadmium Uptake and Translocation Mediated by the Fe^2+^ Transporters OsIRT1 and OsIRT2 in Rice. Soil Sci. Plant Nutr..

[10] Zou R., Wang L., Li Y.C., Tong Z., Huo W., Chi K., Fan H. (2020). Cadmium Absorption and Translocation of Amaranth (*Amaranthus mangostanus* L.) Affected by Iron Deficiency. Environ. Pollut..

[11] Cohen C.K., Fox T.C., Garvin D.F., Kochian L.V. (1998). The Role of Iron-Deficiency Stress Responses in Stimulating Heavy-Metal Transport in Plants. Plant Physiol..

[12] Bao T., Sun L., Sun T., Zhang P., Niu Z. (2009). Iron-Deficiency Induces Cadmium Uptake and Accumulation in *Solanum nigrum* L.. Bull. Environ. Contam. Toxicol..

[13] Connolly E.L., Fett J.P., Guerinot M.L. (2002). Expression of the IRT1 Metal Transporter Is Controlled by Metals at the Levels of Transcript and Protein Accumulation. Plant Cell.

[14] Takahashi R., Ishimaru Y., Senoura T., Shimo H., Ishikawa S., Arao T., Nakanishi H., Nishizawa N.K. (2011). The OsNRAMP1 Iron Transporter Is Involved in Cd Accumulation in Rice. J. Exp. Bot..

[15] Ishimaru Y., Takahashi R., Bashir K., Shimo H., Senoura T., Sugimoto K., Ono K., Yano M., Ishikawa S., Arao T. (2012). Characterizing the Role of Rice NRAMP5 in Manganese, Iron and Cadmium Transport. Sci. Rep..

[16] Sasaki A., Yamaji N., Yokosho K., Ma J.F. (2012). Nramp5 Is a Major Transporter Responsible for Manganese and Cadmium Uptake in Rice. Plant Cell.

[17] Miyadate H., Adachi S., Hiraizumi A., Tezuka K., Nakazawa N., Kawamoto T., Katou K., Kodama I., Sakurai K., Takahashi H. (2011). OsHMA3, a P1B-Type of ATPase Affects Root-to-Shoot Cadmium Translocation in Rice by Mediating Efflux into Vacuoles. New Phytol..

[18] Satoh-Nagasawa N., Mori M., Nakazawa N., Kawamoto T., Nagato Y., Sakurai K., Takahashi H., Watanabe A., Akagi H. (2012). Mutations in Rice (*Oryza sativa*) Heavy Metal ATPase 2 (*OsHMA2*) Restrict the Translocation of Zinc and Cadmium. Plant Cell Physiol..

[19] Takahashi R., Ishimaru Y., Shimo H., Ogo Y., Senoura T., Nishizawa N.K., Nakanishi H. (2012). The OsHMA2 Transporter Is Involved in Root-to-Shoot Translocation of Zn and Cd in Rice. Plant Cell Environ..

[20] Ueno D., Yamaji N., Kono I., Huang C.F., Ando T., Yano M., Ma J.F. (2010). Gene Limiting Cadmium Accumulation in Rice. Proc. Natl. Acad. Sci. USA.

[21] Gao M.Y., Chen X.W., Huang W.X., Wu L., Yu Z.S., Xiang L., Mo C.H., Li Y.W., Cai Q.Y., Wong M.H. (2021). Cell Wall Modification Induced by an Arbuscular Mycorrhizal Fungus Enhanced Cadmium Fixation in Rice Root. J. Hazard. Mater..

[22] Zhu X.F., Wang Z.W., Dong F., Lei G.J., Shi Y.Z., Li G.X., Zheng S.J. (2013). Exogenous Auxin Alleviates Cadmium Toxicity in *Arabidopsis thaliana* by Stimulating Synthesis of Hemicellulose 1 and Increasing the Cadmium Fixation Capacity of Root Cell Walls. J. Hazard. Mater..

[23] Yu H., Wu Y., Huang H., Zhan J., Wang K., Li T. (2020). The Predominant Role of Pectin in Binding Cd in the Root Cell Wall of a High Cd Accumulating Rice Line (*Oryza sativa* L.). Ecotoxicol. Environ. Saf..

[24] Wei W., Peng H., Xie Y., Wang X., Huang R., Chen H., Ji X. (2021). The Role of Silicon in Cadmium Alleviation by Rice Root Cell Wall Retention and Vacuole Compartmentalization under Different Durations of Cd Exposure. Ecotoxicol. Environ. Saf..

[25] Guo X., Luo J., Du Y., Li J., Liu Y., Liang Y., Li T. (2021). Coordination between Root Cell Wall Thickening and Pectin Modification Is Involved in Cadmium Accumulation in *Sedum alfredii*. Environ. Pollut..

[26] Yu R., Jiang Q., Xv C., Li L., Bu S., Shi G. (2019). Comparative Proteomics Analysis of Peanut Roots Reveals Differential Mechanisms of Cadmium Detoxification and Translocation between Two Cultivars Differing in Cadmium Accumulation. BMC Plant Biol..

[27] Lei G.J., Zhu X.F., Wang Z.W., Dong F., Dong N.Y., Zheng S.J. (2014). Abscisic Acid Alleviates Iron Deficiency by Promoting Root Iron Reutilization and Transport from Root to Shoot in *Arabidopsis*. Plant Cell Environ..

[28] Jin C.W., You G.Y., He Y.F., Tang C., Wu P., Zheng S.J. (2007). Iron Deficiency-Induced Secretion of Phenolics Facilitates the Reutilization of Root Apoplastic Iron in Red Clover. Plant Physiol..

[29] Zhu C.Q., Zhang J.H., Zhu L.F., Abliz B., Zhong C., Bai Z.G., Hu W.J., Sajid H., James A.B., Cao X.C. (2018). NH^4+^ Facilitates Iron Reutilization in the Cell Walls of Rice (*Oryza sativa*) Roots under Iron-Deficiency Conditions. Environ. Exp. Bot..

[30] Ye Y.Q., Jin C.W., Fan S.K., Mao Q.Q., Sun C.L., Yu Y., Lin X.Y. (2015). Elevation of NO Production Increases Fe Immobilization in the Fe-Deficiency Roots Apoplast by Decreasing Pectin Methylation of Cell Wall. Sci. Rep..

[31] Ye Y.Q., Luo H.Y., Li M., Zhang J.J., Cao G.Q., Lin K.M., Lin S.Z., Xu S.S. (2019). Potassium Ameliorates Iron Deficiency by Facilitating the Remobilization of Iron from Root Cell Walls and Promoting Its Translocation from Roots to Shoots. Plant Soil.

[32] Zhu X.F., Wu Q., Zheng L., Shen R.F. (2017). NaCl Alleviates Iron Deficiency through Facilitating Root Cell Wall Iron Reutilization and Its Translocation to the Shoot in *Arabidopsis thaliana*. Plant Soil.

[33] Zhu X.F., Lei G.J., Jiang T., Liu Y., Li G.X., Zheng S.J. (2012). Cell Wall Polysaccharides Are Involved in P-Deficiency-Induced Cd Exclusion in *Arabidopsis thaliana*. Planta.

[34] Li S., Zhang Y., Wu Q., Huang J., Shen R.F., Zhu X.F. (2023). Decrease in Hemicellulose Content and Its Retention of Iron Contributes to Phosphorus Deficiency Alleviated Iron Deficiency in *Arabidopsis thaliana*. Plant Sci..

[35] Su Y., Zhang Z., Su G., Liu J., Liu C., Shi G. (2015). Genotypic Differences in Spectral and Photosynthetic Response of Peanut to Iron Deficiency. J. Plant Nutr..

[36] Shi G., Su G., Lu Z., Liu C., Wang X. (2014). Relationship between Biomass, Seed Components and Seed Cd Concentration in Various Peanut (*Arachis hypogaea* L.) Cultivars Grown on Cd-Contaminated Soils. Ecotoxicol. Environ. Saf..

[37] Su G., Li F., Lin J., Liu C., Shi G. (2013). Peanut as a Potential Crop for Bioenergy Production via Cd-Phytoextraction: A Life-Cycle Pot Experiment. Plant Soil.

[38] Su Y., Wang X., Liu C., Shi G. (2013). Variation in Cadmium Accumulation and Translocation among Peanut Cultivars as Affected by Iron Deficiency. Plant Soil.

[39] Tan Z., Li J., Guan J., Wang C., Zhang Z., Shi G. (2023). Genome-Wide Identification and Expression Analysis Reveals Roles of the *NRAMP* Gene Family in Iron/Cadmium Interactions in Peanut. Int. J. Mol. Sci..

[40] Wang C., Wang X., Li J., Guan J., Tan Z., Zhang Z., Shi G. (2022). Genome-Wide Identification and Transcript Analysis Reveal Potential Roles of Oligopeptide Transporter Genes in Iron Deficiency Induced Cadmium Accumulation in Peanut. Front. Plant Sci..

[41] Marschner H., Römheld V. (1994). Strategies of Plants for Acquisition of Iron. Plant Soil.

[42] Su Y., Liu J., Lu Z., Wang X., Zhang Z., Shi G. (2014). Effects of Iron Deficiency on Subcellular Distribution and Chemical Forms of Cadmium in Peanut Roots in Relation to Its Translocation. Environ. Exp. Bot..

[43] He X.L., Fan S.K., Zhu J., Guan M.Y., Liu X.X., Zhang Y.S., Jin C.W. (2017). Iron Supply Prevents Cd Uptake in *Arabidopsis* by Inhibiting *IRT1* Expression and Favoring Competition between Fe and Cd Uptake. Plant Soil.

[44] Kudo K., Kudo H., Kawai S. (2007). Cadmium Uptake in Barley Affected by Iron Concentration of the Medium: Role of Phytosiderophores. Soil Sci. Plant Nutr..

[45] Shao G., Chen M., Wang W., Mou R., Zhang G. (2007). Iron Nutrition Affects Cadmium Accumulation and Toxicity in Rice Plants. Plant Growth Regul..

[46] Yang L., Ding S., Chen X., Cheng Y., Shu P., Wu J., Ma C., Fayyaz P., Zhou J., Deng S. (2025). High Iron Supply Enhances Cadmium Accumulation in the Aerial Tissues by Remodeling the Root Cell Walls in *Populus cathayana*. Ind. Crops Prod..

[47] Li M., Liu C., Zhang D., Wang B., Ding S. (2022). The Influence of Iron Application on the Growth and Cadmium Stress Tolerance of Poplar. Forests.

[48] Parrotta L., Guerriero G., Sergeant K., Cai G., Hausman J.-F. (2015). Target or Barrier? The Cell Wall of Early- and Later-Diverging Plants vs Cadmium Toxicity: Differences in the Response Mechanisms. Front. Plant Sci..

[49] Kanwar P., Bauer P. (2026). Root Cell Wall Plasticity in Iron Homeostasis: An Overlooked Frontier in Plant Nutrition. New Phytol..

[50] Krzesłowska M. (2011). The Cell Wall in Plant Cell Response to Trace Metals: Polysaccharide Remodeling and Its Role in Defense Strategy. Acta Physiol. Plant..

[51] Liu X.X., Zhu X.F., Xue D.W., Zheng S.J., Jin C.W. (2023). Beyond Iron-Storage Pool: Functions of Plant Apoplastic Iron during Stress. Trends Plant Sci..

[52] Zhu X.F., Wang B., Song W.F., Zheng S.J., Shen R.F. (2016). Putrescine Alleviates Iron Deficiency via NO-Dependent Reutilization of Root Cell-Wall Fe in *Arabidopsis*. Plant Physiol..

[53] Wang M., Haris M., Zhang C., Wei T., Zhang L., Laipan M., Guo J. (2025). Cell Wall Polysaccharides Response to Cadmium Stress and Transcriptome Analysis in Tomato (*Solanum lycopersicum*). Plant Sci..

[54] Wang P., Yang B., Wan H., Fang X., Yang C. (2018). The Differences of Cell Wall in Roots between Two Contrasting Soybean Cultivars Exposed to Cadmium at Young Seedlings. Environ. Sci. Pollut. Res..

[55] Cao Q., Xv C., Jiang Q., Wang L., Shi G. (2019). Comparative Transcriptome Analysis Reveals Key Genes Responsible for the Homeostasis of Iron and Other Divalent Metals in Peanut Roots under Iron Deficiency. Plant Soil.

[56] Chen C., Cao Q., Jiang Q., Li J., Yu R., Shi G. (2019). Comparative Transcriptome Analysis Reveals Gene Network Regulating Cadmium Uptake and Translocation in Peanut Roots under Iron Deficiency. BMC Plant Biol..

[57] Sénéchal F., Wattier C., Rustérucci C., Pelloux J. (2014). Homogalacturonan-Modifying Enzymes: Structure, Expression, and Roles in Plants. J. Exp. Bot..

[58] Micheli F. (2001). Pectin Methylesterases: Cell Wall Enzymes with Important Roles in Plant Physiology. Trends Plant Sci..

[59] de Souza A., Hull P.A., Gille S., Pauly M. (2014). Identification and Functional Characterization of the Distinct Plant Pectin Esterases PAE8 and PAE9 and Their Deletion Mutants. Planta.

[60] Mahmood U., Fan Y., Wei S., Niu Y., Li Y., Huang H., Chen Y., Tang Z., Liu L., Qu C. (2021). Comprehensive Analysis of Polygalacturonase Genes Offers New Insights into Their Origin and Functional Evolution in Land Plants. Genomics.

[61] Nagayama T., Tatsumi A., Nakamura A., Yamaji N., Satoh S., Furukawa J., Iwai H. (2022). Effects of Polygalacturonase Overexpression on Pectin Distribution in the Elongation Zones of Roots under Aluminium Stress. AoB Plants.

[62] Ohara T., Takeuchi H., Sato J., Nakamura A., Ichikawa H., Yokoyama R., Nishitani K., Minami E., Satoh S., Iwai H. (2021). Structural Alteration of Rice Pectin Affects Cell Wall Mechanical Strength and Pathogenicity of the Rice Blast Fungus Under Weak Light Conditions. Plant Cell Physiol..

[63] Dwevedi A., Kayastha A.M. (2010). Plant β-Galactosidases: Physiological Significance and Recent Advances in Technological Applications. J. Plant Biochem. Biotechnol..

[64] Katayama-Hirayama K. (1986). Inhibition of the Activities of β-Galactosidase and Dehydrogenases of Activated Sludge by Heavy Metals. Water Res..

[65] Han Y., Sa G., Sun J., Shen Z., Zhao R., Ding M., Deng S., Lu Y., Zhang Y., Shen X. (2014). Overexpression of *Populus euphratica* Xyloglucan Endotransglucosylase/Hydrolase Gene Confers Enhanced Cadmium Tolerance by the Restriction of Root Cadmium Uptake in Transgenic Tobacco. Environ. Exp. Bot..

[66] Zhu X.F., Shi Y.Z., Lei G.J., Fry S.C., Zhang B.C., Zhou Y.H., Braam J., Jiang T., Xu X.Y., Mao C.Z. (2012). *XTH31,* Encoding an in Vitro XEH/XET-Active Enzyme, Regulates Aluminum Sensitivity by Modulating in Vivo XET Action, Cell Wall Xyloglucan Content, and Aluminum Binding Capacity in *Arabidopsis*. Plant Cell.

[67] Zheng S., Ren P., Zhai M., Li C., Chen Q. (2021). Identification of Genes Involved in Root Growth Inhibition Under Lead Stress by Transcriptome Profiling in *Arabidopsis*. Plant Mol. Biol. Rep..

[68] Liu Q., Luo L., Wang X., Shen Z., Zheng L. (2017). Comprehensive Analysis of Rice Laccase Gene (*OsLAC*) Family and Ectopic Expression of *OsLAC10* Enhances Tolerance to Copper Stress in *Arabidopsis*. Int. J. Mol. Sci..

[69] Zhong H., Läuchli A. (1993). Spatial and Temporal Aspects of Growth in the Primary Root of Cotton Seedlings: Effects of NaCl and CaCl_2_. J. Exp. Bot..

[70] Yang J.L., Zhu X.F., Peng Y.X., Zheng C., Li G.X., Liu Y., Shi Y.Z., Zheng S.J. (2011). Cell Wall Hemicellulose Contributes Significantly to Aluminum Adsorption and Root Growth in *Arabidopsis*. Plant Physiol..

[71] Ma J., Liu R., Zhou Z., Zhang Q., Shi G. (2025). Integrative Transcriptomic and Metabolomic Analyses Reveal Crucial Roles of Phenylpropanoid-Derived Coumarin Biosynthesis in the Responses of Peanut to Iron Deficiency. Environ. Exp. Bot..

[72] Livak K.J., Schmittgen T.D. (2001). Analysis of relative gene expression data using real-time quantitative PCR and the 2^−ΔΔCT^ method. Methods.

